# A Novel Trojan Horse Nanotherapy Strategy Targeting the cPKM‐STMN1/TGFB1 Axis for Effective Treatment of Intrahepatic Cholangiocarcinoma

**DOI:** 10.1002/advs.202303814

**Published:** 2023-10-03

**Authors:** Zhi‐Wen Chen, Feng‐Ping Kang, Cheng‐Ke Xie, Cheng‐Yu Liao, Ge Li, Yong‐Ding Wu, Hong‐Yi Lin, Shun‐Cang Zhu, Jian‐Fei Hu, Cai‐Feng Lin, Yi Huang, Yi‐Feng Tian, Long Huang, Zu‐Wei Wang, Shi Chen

**Affiliations:** ^1^ Shengli Clinical Medical College of Fujian Medical University Fuzhou 350001 China; ^2^ Department of Hepatopancreatobiliary Surgery Fujian Provincial Hospital Fuzhou 350001 China; ^3^ Department of Hepatobiliary Surgery and Fujian Institute of Hepatobiliary Surgery Fujian Medical University Union Hospital Fuzhou 350001 China; ^4^ Department of Hepatobiliary Surgery Jinshan Branch of Fujian Provincial Hospital Fuzhou 350001 China; ^5^ Center for Experimental Research in Clinical Medicine Fujian Provincial Hospital Fuzhou 350001 China

**Keywords:** cPKM, intrahepatic cholangiocarcinoma, nanotherapy, STMN1, TGFB1

## Abstract

Intrahepatic cholangiocarcinoma (ICC) is characterized by its dense fibrotic microenvironment and highly malignant nature, which are associated with chemotherapy resistance and very poor prognosis. Although circRNAs have emerged as important regulators in cancer biology, their role in ICC remains largely unclear. Herein, a circular RNA, cPKM is identified, which is upregulated in ICC and associated with poor prognosis. Silencing cPKM in ICC cells reduces TGFB1 release and stromal fibrosis, inhibits STMN1 expression, and suppresses ICC growth and metastasis, moreover, it also leads to overcoming paclitaxel resistance. This is regulated by the interactions of cPKM with miR‐199a‐5p or IGF2BP2 and by the ability of cPKM to stabilize STMN1/TGFB1 mRNA. Based on these findings, a Trojan horse nanotherapy strategy with co‐loading of siRNA against cPKM (si‐cPKM) and paclitaxel (PTX) is developed. The siRNA/PTX co‐loaded nanosystem (Trojan horse) efficiently penetrates tumor tissues, releases si‐cPKM and paclitaxel (soldiers), promotes paclitaxel sensitization, and suppresses ICC proliferation and metastasis in vivo. Furthermore, it alleviates the fibrosis of ICC tumor stroma and reopens collapsed tumor vessels (opening the gates), thus enhancing the efficacy of the standard chemotherapy regimen (main force). This novel nanotherapy provides a promising new strategy for ICC treatment.

## Graphical abstract

This study developed a Trojan horse nanotherapy strategy targeting the cPKM‐STMN1/TGFB1 axis for intrahepatic cholangiocarcinoma (ICC). The si‐cPKM/PTX co‐loaded nanoparticles (Trojan horse) efficiently deliver si‐cPKM and paclitaxel (soldiers) into ICC, promote paclitaxel sensitization, suppress ICC proliferation and metastasis, and reopen collapsed tumor vessels (opening the gates), thus enhancing the efficacy of the standard chemotherapy regimen (main force).

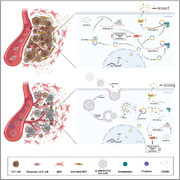

## 1 Introduction

Intrahepatic cholangiocarcinoma (ICC), which originates from the intrahepatic bile duct epithelial cells, is the second most common primary malignant tumor of the liver and accounts for 10%–15% of all primary liver cancers.^[^ ^1^, ^2^ ^]^ Although less prevalent than hepatocellular carcinoma (HCC), ICC is notable for its rapidly increasing global incidence over the past few decades.^[^ ^3^ ^]^ ICC is a fatal disease with a very poor prognosis, and its mortality is stable in most geographic regions, with a 5‐year overall survival (OS) rate of ≈9%.^[^ ^4^ ^]^ As ICC is typically asymptomatic in the early stages, most patients with ICC are diagnosed at an advanced stage. Surgical resection is currently the mainstay of treatment for ICC. Unfortunately, only 20%–40% of patients with potentially operable disease are eligible for surgical resection,^[^ ^3^ ^]^ and caution still needs to be exercised regarding the outcomes after surgical resection. Patents with ICC have a median disease‐free survival rate of 26 months, and the recurrence rate in these patients is as high as 62.2%.^[^ ^5^ ^]^ The current standard treatment for all patients with advanced ICC is systemic chemotherapy with gemcitabine and cisplatin. However, the survival benefits of this chemotherapy regimen are limited, with the median OS duration being <1 year.^[^ ^6^, ^7^ ^]^ In recent years, some studies have attempted to add paclitaxel (PTX) to chemotherapy^[^ ^8^, ^9^ ^]^; however, more evidence is needed to determine whether it is beneficial for patients with ICC. In addition, chemoresistance in ICC is a widely prevalent concern, and it is closely related to unique intracellular molecular mechanisms and apparent extracellular environmental resistance.^[^ ^10^, ^11^ ^]^ Therefore, there is an urgent need to develop new treatment strategies to prolong the survival of patients with ICC.

As is well known, unlike HCC, ICC typically displays an excessive fibrotic response^[^ ^10^ ^]^ and is characterized by a dense tumor stroma with myofibroblasts as the main cell component.^[^ ^12^, ^13^ ^]^ Myofibroblasts in the liver tumor microenvironment are primarily derived from hepatic stellate cells (HSCs) activated by transforming growth factor‐beta 1 (TGFB1).^[^ ^14^, ^15^ ^]^ Activated HSCs can secrete large amounts of collagen and matrix proteins, thus promoting protein cross‐linking and deposition and excessive matrix fibrosis. This creates a dense physical barrier around the tumor that hinders drug penetration; furthermore, it compresses blood vessels, causing collapse and making drug delivery more difficult.^[^ ^10^, ^14^ ^]^ Moreover, the complex molecular mechanisms associated with rapid progression and chemotherapy resistance in ICC cells also explain why ICC treatment is challenging. Therefore, exploring targeted therapies with both intracellular and extracellular effects to efficiently reduce fibrosis and inhibit the malignant behaviors of ICC cells is a new strategy for ICC treatment.

Circular RNAs (circRNAs) are a novel class of non‐coding RNAs (ncRNAs) generated by back‐splicing of precursor mRNAs (pre‐mRNAs), exhibiting complex tissue‐ and stage‐specific expression patterns in the eukaryotic transcriptome.^[^ ^16^ ^]^ CircRNAs play important roles in tumor initiation and progression through multiple mechanisms.^[^ ^17^ ^]^ They can reportedly regulate ICC cell proliferation, metastasis, and drug resistance, thus affecting ICC progression.^[^ ^18^, ^19^ ^]^ However, current studies are largely limited to the regulation of molecular pathways within ICC cells by circRNAs, and little is known about whether circRNAs play a role in the formation and maintenance of the dense tumor stroma in ICC.

In recent years, the emergence of nanomedicine has greatly enriched the means of cancer treatment. Nanoparticles can encapsulate chemotherapeutic drugs and therapeutic small interfering RNAs (siRNAs). Through personalized design and synthesis, they can accumulate in tumors and achieve efficient delivery of chemotherapy drugs and effective interference with target genes.^[^ ^20^ ^]^ In light of the characteristics of ICC, we attempted to develop a nanosystem that can not only reduce ICC fibrosis but also inhibit the malignant behaviors of ICC, thereby achieving effective treatment of ICC on both extracellular and intracellular levels.

In this study, we identified for the first time that cPKM, a circRNA, can both regulate the malignant potential of ICC cells and promote the fibrosis of ICC stroma. cPKM can promote the secretion of TGFB1 by ICC cells, activate HSCs, and induce stromal fibrosis, which compresses blood vessels and hinders drug delivery. In addition, cPKM can upregulate the expression of STMN1, a gene closely related to tumor occurrence, progression, and paclitaxel resistance,^[^ ^21^, ^22^, ^23^ ^]^ thus enhancing the proliferation, metastasis, and paclitaxel resistance of ICC cells. Mechanistically, cPKM increases the stability of STMN1 and TGFB1 mRNAs by binding to the m^6^A reader IGF2BP2. In addition, cPKM can act as a sponge to absorb miR‐199a‐5p, thus upregulating STMN1 expression. As ICC is like the ancient city of Troy, with dense stroma on the outside and aberrantly expressed cancer‐promoting molecules on the inside, we devised a Trojan horse‐resembling nanotherapy strategy by developing a dual‐vector nanosystem that co‐delivers si‐cPKM and paclitaxel. This nanosystem, functioning as a Trojan horse, can safely and effectively co‐deliver si‐cPKM and paclitaxel to ICC cells. We identified that silencing cPKM in ICC cells with a siRNA inhibited the expression of STMN1, weakened the proliferation and metastasis ability of ICC cells, and sensitized them to paclitaxel. Furthermore, inhibiting TGFB1 release reduced tumor stromal fibrosis, opened blood vessels, and enhanced the efficacy of the standard chemotherapy regimen, thereby significantly improving the survival of the orthotopic tumor‐bearing mice. Our pre‐clinical evaluation of this Trojan horse‐resembling nanotherapy provides critical insights into the development of this siRNA/paclitaxel co‐delivery nanosystem as a promising new strategy for ICC treatment.

## 2 Results

### 2.1 cPKM is Upregulated in ICC Tissues and a Higher cPKM Expression is Associated with Worse Prognosis

**Figure 1:**
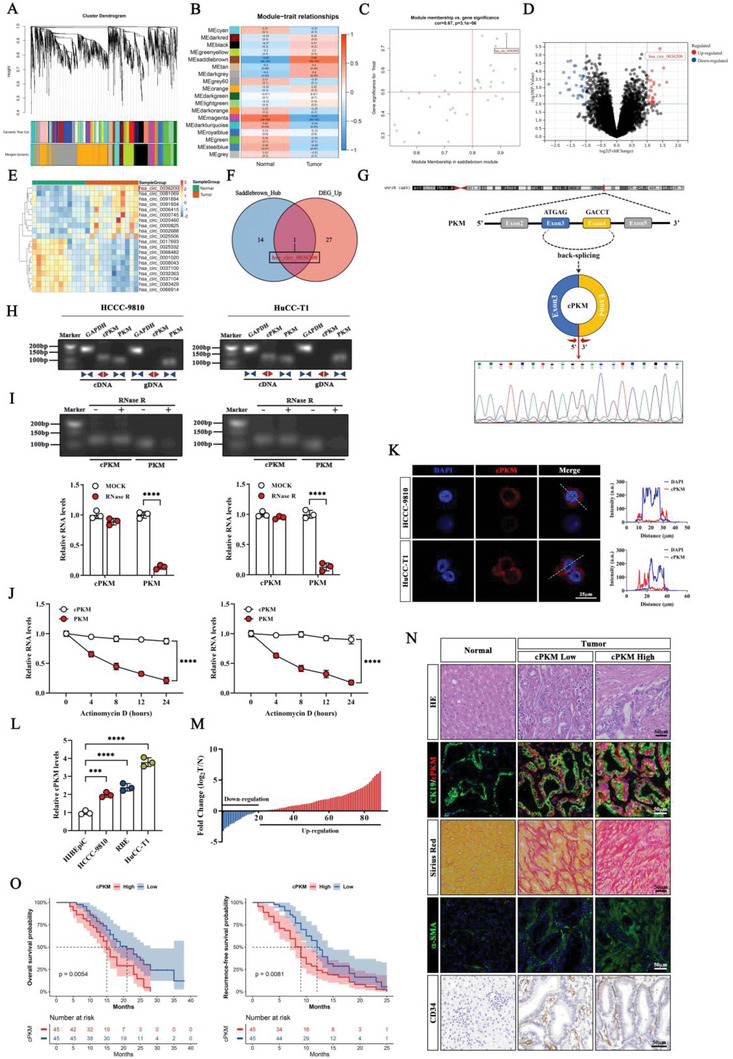
Screening of circRNAs in ICC and characterization of cPKM and its clinical significance in patients with ICC A) Co‐expressed gene dendrogram clustering diagram. Each branch represents a gene, and each color at the bottom represents a co‐expression module. B) Heatmap of correlations of modules with clinical traits. Each cell contains the corresponding correlation and *p*‐value. C) Scatter plot of gene significance (GS) versus module membership (MM) in Saddlebrown modules. D) Volcano plot (|logFC| >1, *P* value < 0.01) and E) heatmap (top 10 significantly up‐ or downregulated circRNAs) of E) differentially expressed circRNAs in ICC and normal tissues. F) Venn diagram of overlapping circRNAs. G) A schematic of the genomic region of cPKM and its cyclization (upper). Back‐splicing junction of cPKM identified by Sanger sequencing (lower). H) Agarose gel electrophoresis images show the amplification of cPKM and PKM in both complementary DNA (cDNA) and genomic DNA (gDNA) from HCCC‐9810 cells and HuCC‐T1 cells using convergent (blue) and divergent (red) primers. I) Agarose gel electrophoresis and qRT‐PCR were used to analyze the expression of cPKM and PKM mRNAs after RNase R treatment (*n* = 3). J) Relative levels of cPKM and PKM mRNAs after actinomycin D treatment were detected by qRT‐PCR at the indicated time points. (*n* = 3). K) Subcellular localization of cPKM was detected by fluorescence in situ hybridization (FISH). cPKM and DAPI signals are shown along the lines. L) Relative levels of cPKM in normal human bile duct epithelial cells and ICC cells were determined by qRT‐PCR (*n* = 3). M) The level of cPKM in ICC tissues relative to paired normal tissues was detected by qRT‐PCR. (N) Representative images of H&E staining, CK19 (IF), and cPKM (FISH) co‐staining, Sirius red staining, α‐SMA (IF) staining, and CD34 (IHC) staining in the indicated groups. O) Kaplan–Meier survival analysis of the association of the cPKM level with overall survival (OS) and recurrence‐free survival (RFS) in 90 patients with ICC. The median cPKM level was used as the cut‐off value. *n* indicates biological replicates. Data are the mean ± SD ***p < 0.001, ****p < 0.0001. Two‐way (I, J) or one‐way (L) ANOVA and O) log‐rank test (O).

To explore the abundant and differentially expressed circRNAs in ICC, we collected five pairs of ICC tissues and paired normal tissues for circRNA microarray analysis. In addition, we included the public circRNA microarray data for seven pairs of ICC tissues and paired normal tissues from GSE181523 in Gene Expression Omnibus (GEO). After removing the batch effect from both datasets (Figure S1A, Supporting Information), we merged the two datasets for subsequent analysis. First, we performed the weighted gene co‐expression network analysis (WGCNA), with the soft threshold power set to *β* = 20, and we constructed a scale‐free network evaluation with coefficient *R* ^2^ = 0.85 and slope = −1.29 (Figure S1B,C, Supporting Information). We used the dynamic tree‐cut method for module identification and merged similar modules to obtain 18 modules (**Figure** **1**A). The correlation between clinical features and modules is shown in the heatmap (Figure 1B), and we found the Saddlebrown module to be the most correlated with the tumor (correlation coefficient = 0.66, *p* < 0.001). In the Saddlebrown module, we further set the cutoff criteria for gene significance (GS) and module membership (MM, MM > 0.8, and GS > 0.5) and screened 15 candidate hub circRNAs (Figure 1C). In addition, we performed differential expression analysis and identified 59 differentially expressed circRNAs (|logFC| > 1, *p*‐value < 0.01), including 28 upregulated and 31 downregulated circRNAs, as shown in the volcano plot (Figure 1D). The top 10 significantly upregulated or downregulated circRNAs were visualized in a heatmap (Figure 1E). Previous studies have shown that some circRNAs are abnormally overexpressed in tumors and play important roles in tumor progression.^[^ ^24^, ^25^ ^]^ Therefore, we focused on the significantly upregulated circRNAs in ICC and identified hsa_circ_0036200 from the intersection of the significantly upregulated circRNAs and candidate hub circRNAs in the Saddlebrown module (Figure 1F, Table S1, Supporting Information). Hsa_circ_0036200, which we have referred to as cPKM hereafter, is derived from back‐splicing of exons 2 and 3 of the human PKM (pyruvate kinase M1/2) gene on chr15q23 and contains 259 nucleotides (Figure 1G).

To confirm the circular characteristic of cPKM, we designed divergent and convergent primers, and cPKM could be amplified by divergent primers in cDNA from ICC cells (Figure 1H). Sanger sequencing further confirmed the back‐splicing junction (BSJ) site of cPKM in the amplified product (Figure 1G). cPKM showed evident resistance to RNase R treatment (Figure 1I), and in ICC cells treated with the transcription inhibitor actinomycin D, cPKM was found to be more stable than its corresponding linear transcript (Figure 1J). Moreover, RNA fluorescent in situ hybridization (FISH) showed that cPKM was mainly localized in the cytoplasm (Figure 1K). We further verified the expression levels of cPKM in cell lines using qRT‐PCR, and the results showed that all the tested ICC cell lines (HCCC‐9810, RBE, and HuCC‐T1) had significantly upregulated cPKM expression than human intrahepatic bile duct epithelial cells (HIBEpiC, Figure 1L). In addition, cPKM expression was found to be higher in most ICC tissues than in paired normal tissues in 90 patients with ICC (Figure 1M). We examined the expression of cPKM in the tissues using RNA‐FISH, and consistent with the results of qRT‐PCR, cPKM expression was significantly higher in ICC tissues than in normal tissues (Figure 1N). Interestingly, we also found that ICC tissues with high cPKM expression had more significant fibrosis, higher alpha‐smooth muscle actin (α‐SMA) abundance, and more collapsed blood vessels (Figure 1N). Then, we examined the association between cPKM expression and clinicopathological characteristics of patients with ICC. Higher cPKM expression levels were significantly associated with elevated serum carbohydrate antigen 19‐9 (CA19‐9) levels, greater presence of cirrhosis and lymph node metastases, larger tumor size, poorer tumor differentiation, and more advanced tumor stages (**Table** **1**). Furthermore, the Kaplan–Meier survival analysis showed that patients with ICC having increased cPKM expression levels had worse overall survival (OS) and recurrence‐free survival (RFS, Figure 1O). Taken together, these data show the upregulation of cPKM in ICC and its clinical significance in patients with ICC.

**Table 1:** Baseline characteristics of 90 patients with ICC according to cPKM expression levels

Characteristic	cPKM low‐expression	cPKM high‐expression	*p‐*value
	*n* = 45	*n* = 45	
Sex			0.832
male	24(53.3)	25(55.6)	
female	21(46.7)	20(44.4)	
Age			0.671
<60	19(42.2)	21(46.7)	
≥60	26(57.8)	24(53.3)	
BMI			0.499
<25	32(71.1)	29(64.4)	
≥25	13(28.9)	16(35.6)	
HBsAg			0.612
negative	34(75.6)	36(80.0)	
positive	11(24.4)	9(20.0)	
Cirrhosis	8(17.8)	19(42.2)	0.011
Hepatolithisasis	11(24.4)	10(22.2)	0.803
CA19‐9, UI mL^−1^	85.9(47.3–146.3)	133.5(66.3–230.7)	0.005
Differentiation			0.002
well	5(11.1)	2(4.4)	
moderate	31(68.9)	18(40.0)	
poor	9(20.0)	25(55.6)	
Tumor number			0.18
single	42(93.3)	38(84.4)	
multiple	3(6.7)	7(15.6)	
Tumor size, cm	4.5(3.5–5.4)	5.7(4.6–6.4)	0.001
Lymph node metastasis	25(55.6)	40(88.9)	<0.001
AJCC			0.015
I	8(17.8)	1(2.2)	
II	11(24.4)	7(15.6)	
III	26(57.8)	37(82.2)	

### 2.2 cPKM Promotes Proliferation and Metastasis of ICC Cells and Induces Myofibroblast Activation in HSCs

To explore the potential biological functions of cPKM in ICC, we constructed three small interfering RNAs (siRNAs) targeting the BSJ of cPKM (Figure S2A, Supporting Information) and cPKM overexpression plasmids. Since si‐cPKM‐2 was the most effective in HuCC‐T1 cells (Figure S2B, Supporting Information), si‐cPKM‐2 was chosen for silencing cPKM in the following experiments. In addition, the overexpression efficiency of the cPKM‐expressing plasmid in HCCC‐9810 cells was verified (Figure S2C), and linear PKM mRNA expression levels in both HuCC‐T1 cells and HCCC‐9810 cells were not affected (Figure S2B,C).

**Figure 2:**
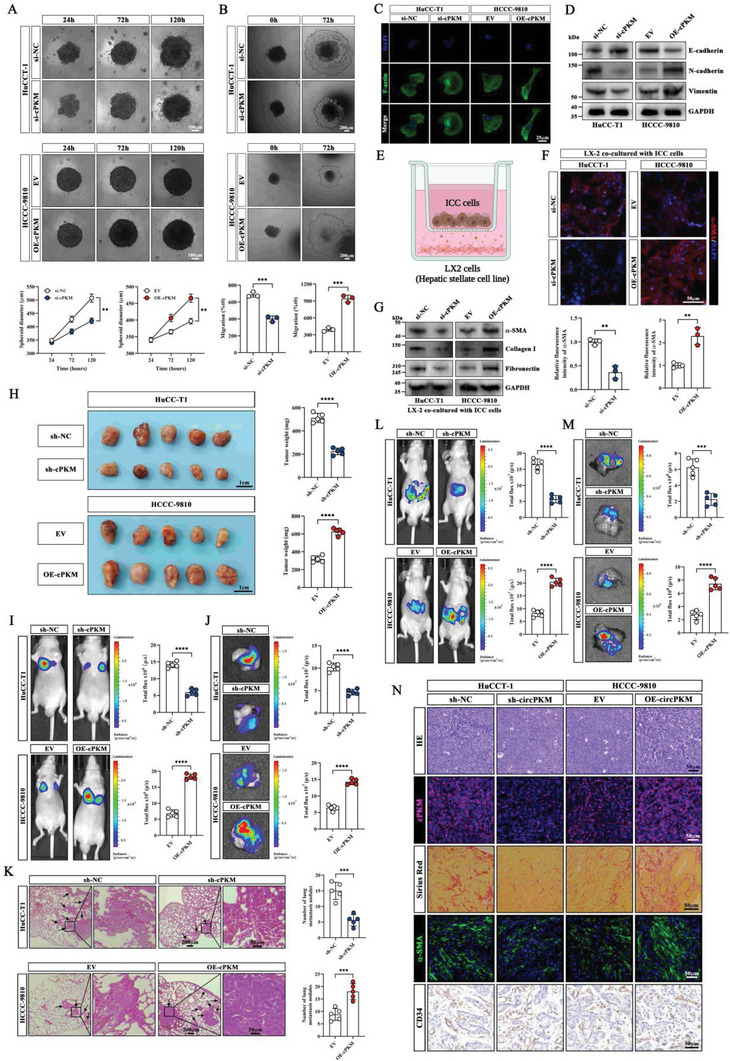
The biological effects of altered cPKM expression on regulating the malignancy of ICC cells. A) Representative images of knocked down cPKM (KD‐cPKM) or overexpressed cPKM (OE‐cPKM) ICC microtumor spheroids and quantification of spheroid diameters at different time points (*n* = 3). B) Representative images of the migration and the relative migration area of KD‐cPKM or OE‐cPKM ICC microtumor spheroids at different time points (*n* = 3). C) F‐actin (green) staining of the indicated ICC cells by phalloidin. D) Western blot analysis of E‐cadherin, N‐cadherin, and vimentin in KD‐cPKM or OE‐cPKM ICC cells. E) A schematic of co‐culturing ICC cells with LX‐2 cells (created using BioRender.com). F) Representative images of α‐SMA immunofluorescence and relative fluorescence intensity in LX‐2 cells after co‐culture with KD‐cPKM or OE‐cPKM ICC cells (*n* = 3). G) Western blot analysis of α‐SMA, fibronectin, and COL1A1 in LX‐2 cells after co‐culture with the indicated ICC cells. H) Images and weights of subcutaneous xenografts of KD‐cPKM and OE‐cPKM ICC cells (*n* = 5). I,J) Representative images and statistical analysis of I) in vivo and J) ex vivo lung bioluminescence in the indicated lung metastasis models (*n* = 5). K) H&E staining and quantification of lung metastases (*n* = 5). L,M) Representative images and statistical analysis of L) in vivo and M) ex vivo liver bioluminescence in the indicated orthotopic tumor models (*n* = 5). N) Representative images of H&E staining, cPKM (FISH) staining, Sirius red staining, α‐SMA (IF) staining, and CD34 (IHC) staining in the indicated orthotopic tumors. *n* indicates biological replicates (A, B, and F) or individual mice (H–L). Data are the mean ± SD **p < 0.01, ***p < 0.001, ****p < 0.0001. Two‐way ANOVA (A) and unpaired two‐tailed Student's *t*‐tests (B, F, H‐L).

We first performed 3D microtumor spheroid formation and growth assays to evaluate the impact of manipulated cPKM expression on ICC growth. The results showed that cPKM knockdown inhibited the growth of HuCC‐T1 microtumor spheroids, whereas cPKM overexpression promoted the growth of HCCC‐9810 tumor spheroids (**Figure** **2**A). In the 3D microtumor spheroid‐based migration assay, we found that cPKM knockdown attenuated the migration ability of HuCC‐T1 microtumor spheroids, whereas cPKM overexpression enhanced the migration ability of HCCC‐9810 microtumor spheroids (Figure 2B). Cytoskeletal reorganization is a characteristic of epithelial–mesenchymal transition (EMT).^[^ ^26^ ^]^ Using F‐actin staining by phalloidin, we found that cPKM knockdown reduced the formation of filamentous pseudopods in ICC cells, whereas increased formation of filamentous pseudopods was observed after cPKM overexpression (Figure 2C). In addition, Western blot analysis showed that cPKM knockdown downregulated N‐cadherin and vimentin expression but promoted E‐cadherin expression in ICC cells; conversely, the opposite results were obtained with cPKM overexpression (Figure 2D). HSCs are reportedly a major contributor to liver fibrosis.^[^ ^27^ ^]^ HSCs are in a resting state in healthy livers; however, when stimulated by inflammation or tumor cells, they can transdifferentiate into myofibroblasts associated with connective tissue proliferative response and tumor development.^[^ ^14^, ^15^ ^]^ ICC is a highly fibrous proliferative tumor with a dense stroma rich in α‐SMA‐positive myofibroblasts.^[^ ^28^ ^]^ Based on the finding that cPKM expression levels are correlated with the degree of fibrosis, we speculated that ICC cells with high cPKM expression promote myofibroblast activation in HSCs. To test this hypothesis, we established a co‐culture model of ICC cells and HSCs, as shown in Figure 2E. Immunofluorescence (IF) staining for α‐SMA confirmed that knockdown (KD)‐cPKM ICC cells caused less myofibroblast activation in HSCs, whereas overexpressing (OE)‐cPKM ICC cells induced more myofibroblast activation in HSCs (Figure 2F). Moreover, Western blot analysis also showed that the expression of hepatic stellate cell activation markers (α‐SMA, fibronectin, and COL1A1) decreased after co‐culture with KD‐cPKM ICC cells and increased after co‐culture with OE‐cPKM ICC cells (Figure 2G).

To determine the biological function of cPKM in vivo, subcutaneous xenograft tumors were generated in nude mice. We observed that the subcutaneous tumor weight was lower in the KD‐cPKM group but higher in the OE‐cPKM group (Figure 2H). Next, we established a lung metastasis model; we monitored metastasis using the IVIS imaging system and quantified the number of lung metastasis nodules by H&E staining. The results showed reduced luciferase activity in lung metastases and fewer lung metastasis nodules in the KD‐cPKM group, and the opposite trends were noted in the OE‐cPKM group (Figure 2K). To simulate the ICC tumor microenvironment, we established an orthotopic model by intrahepatic injection of ICC cells. The in vivo IVIS imaging results showed that cPKM overexpression promoted the growth of ICC tumors in the mouse liver. In addition, surprisingly, we observed more pronounced fibrosis, higher α‐SMA abundance, and more vascular collapse, which mimicked the tumor microenvironments of patients having ICC with high cPKM expression. In contrast, the growth of intrahepatic ICC tumors was inhibited in the KD‐cPKM group which showed reduced fibrosis and α‐SMA abundance and more vascular opening (Figure 2N). Collectively, our experimental results suggest that cPKM exerts both intracellular and extracellular biological effects: it promotes the proliferation and metastasis of ICC cells and induces myofibroblast activation in HSCs, which promotes the proliferation of ICC tumor stromal fibers, and causes intratumor microvascular collapse.

### 2.3 The cPKM‐IGF2BP2‐STMN1/TGFB1 Complex Stabilizes STMN1/TGFB1 mRNA

**Figure 3:**
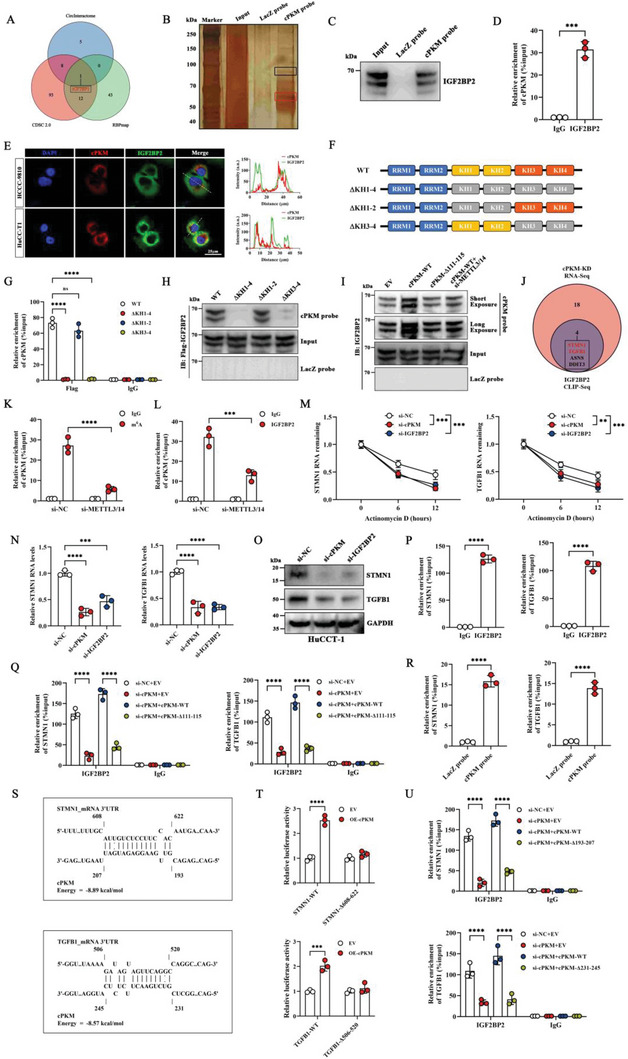
cPKM acts as a bridge to prompt IGF2BP2 to bind and stabilize STMN1/TGFB1 mRNA A) Venn diagram showing the overlap of potential binding proteins of cPKM. B) Cell lysate was incubated with the cPKM probe, which was followed by the RAP assay to pull down potential cPKM‐binding proteins and subsequent visualization by silver staining. C) The association of cPKM with IGF2BP2 was verified by Western blot analysis. D) The RIP assay showing the association of IGF2BP2 with cPKM (*n* = 3). E) Subcellular localization of cPKM (red) and IGF2BP2 (green) was detected by FISH and immunofluorescence. cPKM and IGF2BP2 signals are shown along the lines. F) A schematic shows the RNA‐binding domain within the IGF2BP2 protein and a summary of the IGF2BP2 variants. The gray box is the KH domain after the mutation of GxxG to GEEG. G) The RIP assay showing the enrichment level of cPKM in the indicated groups (*n* = 3). H) The associations of cPKM with IGF2BP2 or IGF2BP2 variants were verified by Western blot analysis. I) Protein levels of IGF2BP2 pulled down by the cPKM probe after the overexpression of cPKM and cPKM mutants was verified by Western blot analysis. J) A Venn diagram showing possible target genes of cPKM and IGF2BP2. K) The MeRIP assay to determine the level of m^6^A modification in cPKM after METTL3/14 knockdown (*n* = 3). L) The RIP assay to determine the level of cPKM enriched by IGF2BP2 after METTL3/14 knockdown (*n* = 3). M) The mRNA levels of STMN1 and TGFB1 were analyzed by qRT‐PCR in HuCC‐T1 cells with cPKM or IGF2BP2 knockdown after ActD treatment for different durations (*n* = 3). N,O) Analysis of mRNA and protein levels of STMN1 and TGFB1 in cPKM or IGF2BP2 knockdown HuCC‐T1 cells by N) qRT‐PCR and O) Western blot, respectively (*n* = 3). P) The RIP assay showing the association of IGF2BP2 with STMN1 and TGFB1 mRNAs (*n* = 3). Q) RIP assay showing the levels of STMN1 or TGFB1 mRNA enriched by IGF2BP2 in the indicated groups (*n* = 3). R) The RAP assay showing the association of cPKM with STMN1 and TGFB1 mRNAs (*n* = 3). S) Potential interaction sites of cPKM with STMN1 or TGFB1 mRNA as predicted by IntaRNA. T) The relative luciferase activity of the indicated luciferase reporter vectors after cPKM overexpression (*n* = 3). U) The RIP assay showing the level of STMN1 or TGFB1 mRNA enriched by IGF2BP2 in the indicated groups (*n* = 3). *n* indicates biological replicates. Data are the mean ± SD **p < 0.01, ***p < 0.001, ****p < 0.0001. Unpaired two‐tailed Student's *t*‐tests (D, P, R) and two‐way (G, K‐M, Q, T, U) or one‐way (N) ANOVA.

CircRNAs can participate in molecular mechanisms regulating tumor progression by interacting with proteins,^[^ ^29^ ^]^ and we employed multiple online tools, such as CircInteractome (https://circinteractome.irp.nia.nih.gov/), CDSC 2.0 (http://gb.whu.edu.cn/CSCD2/), and RBPmap (http://rbpmap.technion.ac.il/), to identify proteins that potentially interact with cPKM. We found that IGF2BP2 was included in these three databases (**Figure** **3**A, Table S2, Supporting Information). Next, we performed the RNA antisense purification (RAP) assay using a specific biotin‐labeled cPKM probe and a control probe. The cPKM probe efficiently enriched cPKM but not PKM (Figure S3A, Supporting Information). After silver staining, a sense‐specific band at ∼65 kDa (red box) was observed in the cPKM probe group (Figure 3B). Subsequently, the interaction between cPKM and IGF2BP2 was confirmed by Western blot analysis and the RNA immunoprecipitation (RIP) assay (Figure 3C,D). In addition, RNA‐FISH combined with IF showed that cPKM and IGF2BP2 co‐localized in the cytoplasm (Figure 3E). The KH structural domain of IGF2BPs, particularly the GxxG motif, is reportedly essential for the interaction between IGF2BPs and RNAs.^[^ ^30^, ^31^ ^]^ To explore whether the KH structural domain of IGF2BP2 is essential for the interaction with cPKM, we constructed multiple IGF2BP2 variants with GxxG mutated to GEEG in the KH structural domain (Figure 3F). Mutations in the KH3–4 double domain markedly reduced the interaction of IGF2BP2 with cPKM (Figure 3G,H). Previous studies have shown that IGF2BP proteins preferentially bind with the “UGGAC” consensus sequence,^[^ ^30^ ^]^ and we identified a potential IGF2BP binding region in the cPKM sequence (blue background, Figure S3C, Supporting Information). Next, we constructed a cPKM mutant (cPKM‐Δ111–115) by mutating “UGGAC” to “GUUCA” (yellow background, Figure S3C, Supporting Information). The RAP assay showed that the enrichment level of IGF2BP2 was increased in the cPKM‐WT overexpression group but not in the cPKM‐Δ111–115 overexpression group (Figure 3I). The “UGGAC” sequence contains the N6‐methyladenosine (m^6^A) core motif “GGAC.” To investigate whether the interaction of cPKM with IGF2BP2 is dependent on m^6^A modification, we performed methylated RNA immunoprecipitation (MeRIP) and found that m^6^A was significantly enriched in cPKM (Figure 3K). Knockdown of METTL3 and METTL14 significantly reduced the level of m^6^A modification in cPKM (Figure 3K) and impaired the interaction between cPKM and IGF2BP2 (Figure 3I,L). Our data suggest that IGF2BP2 binds to the “UGGAC” sequence of cPKM in an m^6^A‐dependent manner via the KH3–4 double domain.

The expression of cPKM was not significantly altered after IGF2BP2 knockdown (Figure S3B,D, Supporting Information). cPKM knockdown or overexpression resulted in little change in the IGF2BP2 protein level (Figure S3E, Supporting Information), suggesting that cPKM was not involved in IGF2BP2 post‐translational modifications. As IGF2BP2 could maintain RNA stability,^[^ ^30^, ^32^ ^]^ we wondered whether cPKM was regulating some unknown downstream targets by interacting with IGF2BP2. We performed RNA sequencing analysis after cPKM knockdown in HuCC‐T1 cells and found that 22 genes showed significant expression changes (|logFC| > 1.5, *p*‐value < 0.01). As IGF2BP2 preferentially binds to the 3ʹ untranslated region (UTR) of the target RNA,^[^ ^30^, ^32^ ^]^ we screened the ENCORI database (https://starbase.sysu.edu.cn/) based on CLIP‐seq assays for IGF2BP2‐binding 3ʹUTRs. Among the differentially expressed genes, we identified four potential targets (STMN1, TGFB1, ASNS, and DDIT3, Figure 3J, Table S3, Supporting Information). As verified by qRT‐PCR, the mRNA stability and expression levels of STMN1 and TGFB1 were significantly reduced after cPKM or IGF2BP2 knockdown (Figure 3M,N), whereas those of ASNS and DDIT3 did not significantly change (Figure S3F,G, Supporting Information). Therefore, we focused on STMN1 and TGFB1.

It was further confirmed by Western blot analysis that cPKM or IGF2BP2 knockdown reduced the protein levels of STMN1 and TGFB1 (Figure 3O). Subsequently, RIP assays confirmed that IGF2BP2 interacted with STMN1 and TGFB1 mRNAs (Figure 3P). Interestingly, although cPKM knockdown reduced the binding of IGF2BP2 with STMN1 and TGFB1 mRNAs (Figure 3Q), it did not affect m^6^A modification levels in STMN1 and TGFB1 mRNAs (Figure S3H, Supporting Information). In addition, the association of IGF2BP2 with STMN1 and TGFB1 mRNAs could be restored by wild‐type cPKM (cPKM‐WT) but not by mutated cPKM (cPKM‐Δ111–115, Figure 3Q), suggesting that cPKM plays an important role in the interaction of IGF2BP2 with STMN1 and TGFB1 mRNAs through the mutated region. RAP assays showed that both STMN1 and TGFB1 mRNAs were significantly enriched in the cPKM probe set (Figure 3R). Then, we identified interaction sites of cPKM with STMN1 and TGFB1 mRNA 3ʹUTRs using the IntaRNA program (http://rna.informatik.uni‐freiburg.de/IntaRNA), and two putative interaction regions were predicted, respectively (Figure 3S and Figure S3I, Supporting Information). To further identify the putative interaction regions of cPKM with STMN1 and TGFB1 mRNA 3ʹUTRs that are essential for STMN1 and TGFB1 mRNA stabilization, we further constructed luciferase reporter plasmids containing wild‐type 3ʹUTR (STMN1‐WT and TGFB1‐WT) or mutant 3ʹUTR (STMN1‐Δ 608–622, STMN1‐Δ628–643, TGFB1‐Δ506–520, and TGFB1‐Δ557–574). The results showed that cPKM overexpression resulted in increased luciferase activity in STMN1‐WT, STMN1‐Δ628–643, TGFB1‐WT, and TGFB1‐Δ557–574 constructs but not in STMN1‐Δ608–622 and TGFB1‐Δ506–520 constructs (Figure 3T and Figure S3J, Supporting Information). Based on these results, we constructed two cPKM mutants (cPKM‐Δ193–207 and cPKM‐Δ231–245), wherein cPKM‐Δ193–207 was a mutation of the complementary region to STMN1 mRNA 3ʹUTR 608–622 nt in cPKM and cPKM‐Δ231–245 was a mutation of the complementary region to TGFB1 mRNA 3ʹUTR 506–520 nt in cPKM. Using the RIP assay, we found that cPKM knockdown reduced the binding of IGF2BP2 to STMN1 or TGFB1 mRNA, and the association could be restored by cPKM‐WT but not by cPKM‐Δ193–207 or cPKM‐Δ231–245 (Figure 3U). It indicated that the sequence complementary binding of cPKM to STMN1/TGFB1 mRNA 3ʹUTR was important for the binding of IGF2BP2 with STMN1/TGFB1 mRNA. Based on the above evidence, we inferred that cPKM can bind with IGF2BP2 and STMN1/TGFB1 mRNA 3ʹUTR to form RNA–protein complexes and that it promoted the interaction between IGF2BP2 and STMN1/TGFB1 mRNA, which in turn enhanced the mRNA stability of STMN1/TGFB1.

### 2.4 cPKM Ats as an miRNA Sponge for miR‐199a‐5p and Upregulates STMN1 Expression

**Figure 4:**
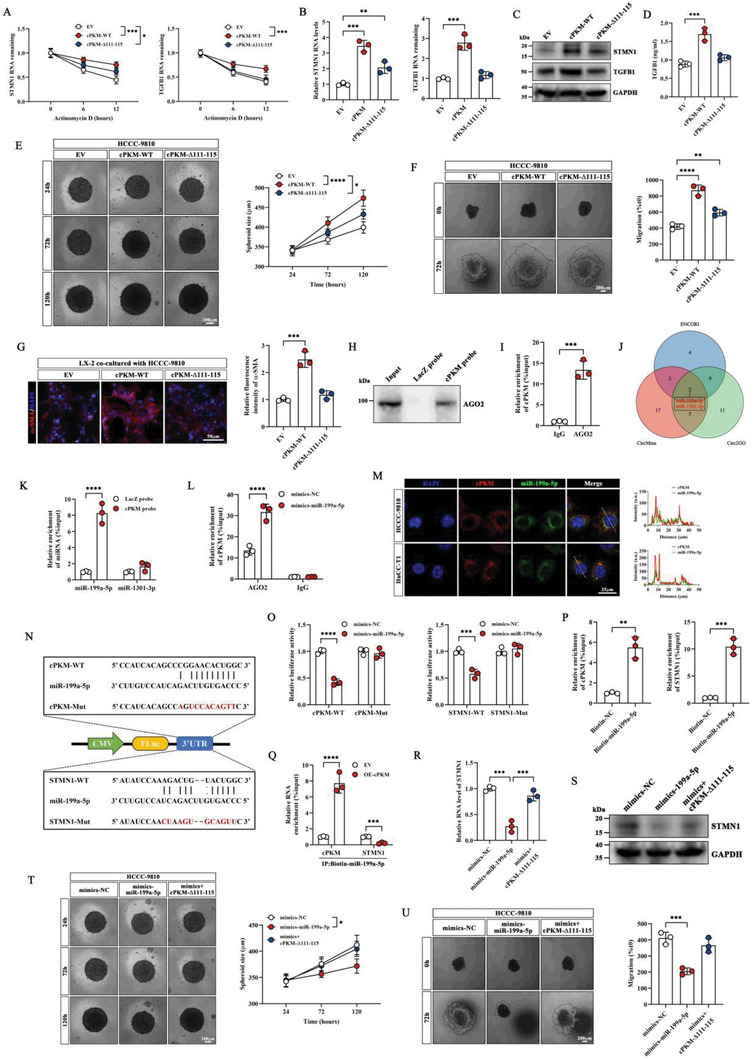
cPKM acts as a miR‐199a‐5p sponge and promotes STMN1 expression A) The mRNA levels of STMN1 and TGFB1 were analyzed by qRT‐PCR in HCCC‐9810 cells overexpressing cPKM or cPKM variants after ActD treatment at different timepoints (*n* = 3). (B, C) The mRNA and protein levels of STMN1 and TGFB1 in HCCC‐9810 cells overexpressing cPKM or cPKM variants as estimated using B) qRT‐PCR and C) Western blot analysis, respectively (*n* = 3). D) The concentration of TGFB1 in the supernatant of HCCC‐9810 cells overexpressing cPKM or cPKM variants was determined using ELISA (*n* = 3). E) Representative images of the indicated HCCC‐9810 microtumor spheroids and quantification of spheroids diameter at different time points (*n* = 3). F) Representative images of migration and the relative migration areas of the indicated HCCC‐9810 microtumor spheroids at different time points (*n* = 3). G) Representative images of immunofluorescence and the relative fluorescence intensity of α‐SMA in LX‐2 cells after co‐culture with the indicated HCCC‐9810 cells (*n* = 3). H) The association of cPKM with AGO2 was verified by the RAP assay combined with Western blot analysis. I) The RIP assay showing the association of AGO2 with cPKM (*n* = 3). J) A Venn diagram showing overlapping potential target miRNAs of cPKM. K) The levels of miR‐199a‐5p and miR‐1301‐3p enriched by cPKM probes (*n* = 3). L) The AGO2‐RIP assay showing the enrichment levels of cPKM after transfection of miR‐199a‐5p mimics (*n* = 3). M) Subcellular localization of cPKM (red) and miR‐199a‐5p (green) was detected by FISH. cPKM and miR‐199a‐5p signals are shown along the arrows. N) A schematic of luciferase reporter vectors for cPKM‐WT, cPKM‐Mut, STMN1‐WT, and STMN1‐Mut. O) The relative luciferase activity of the indicated luciferase reporter vectors after transfection of miR‐199a‐5p mimics (*n* = 3). P) Enrichment of cPKM and STMN1 mRNA levels by biotin‐labeled miR‐199a‐5p mimics (*n* = 3). Q) Enrichment of cPKM and STMN1 mRNA levels by biotin‐labeled miR‐199a‐5p mimics after cPKM overexpression (*n* = 3). R,S) STMN1 mRNA and protein levels in the indicated HCCC‐9810 cells were quantified by R) qRT‐PCR and S) Western blot analysis, respectively (*n* = 3). T) Representative images of the indicated HCCC‐9810 microtumor spheroids and spheroid diameters at different time points (*n* = 3). U) Representative images of the indicated HCCC‐9810 microtumor spheroid migration and the relative migration area at different time points (*n* = 3). *n* indicates biological replicates. Data are the mean ± SD *p < 0.05, **p < 0.01, ***p < 0.001, ****p < 0.0001. Two‐way (A, E, K, L, O, Q, T) or one‐way (B, D, F, G, R, U) ANOVA and unpaired two‐tailed Student's *t*‐tests (I,P).

To further confirm if cPKM stabilizes STMN1/TGFB1 mRNA by interacting with IGF2BP2, we overexpressed cPKM‐WT and cPKM‐Δ111–115 in ICC cells. We found that cPKM‐WT stabilized STMN1 and TGFB1 mRNAs and increased STMN1 and TGFB1 expression; conversely, cPKM‐Δ111–115 had no effect on TGFB1 expression but partially enhanced STMN1 expression (**Figure** **4**A–C). TGFB1 can reportedly be synthesized and secreted by tumor cells.^[^ ^33^ ^]^ We further examined the level of TGFB1 in ICC cell culture supernatants by ELISA and found that cPKM‐WT increased the level of TGFB1 but cPKM‐Δ111–115 did not (Figure 4D). In vitro biological function assays showed that cPKM‐Δ111–115 partially enhanced ICC cell proliferation and migration but failed to promote HSC myofibroblast activation (Figure 4E–G). Previous studies have shown that STMN1 plays an important role in tumor proliferation and metastasis^[^ ^21^, ^23^ ^]^ and that TGFB1 is an important cytokine for myofibroblast activation in HSCs.^[^ ^14^, ^34^, ^35^ ^]^ Based on the abovementioned findings, we hypothesized that cPKM increased the expression and secretion of TGFB1 to promote HSC myofibroblast activation by interacting with IGF2BP2. Besides, cPKM may regulate the expression of STMN1 by interacting with IGF2BP2 and via other mechanisms without the involvement of IGF2BP2, thus promoting the proliferation and metastasis of ICC cells.

CircRNAs can encode proteins for biological functions^[^ ^36^ ^]^; however, our analysis using the circRNADb database (http://reprod.njmu.edu.cn/cgi‐bin/circrnadb/circRNADb.php) showed that cPKM has a relatively low potential to encode proteins (Figure S4A, Supporting Information). In addition, our analysis with the Coding Potential Calculator 2 (CPC2) algorithm (http://cpc2.gao‐lab.org/) also indicated that cPKM cannot encode proteins (Figure S4B, Supporting Information). According to previous studies, circRNAs localized in the cytoplasm are usually associated with miRNA sponge interactions,^[^ ^37^, ^38^ ^]^ and AGO2 protein has an important role in the functioning of circRNA.^[^ ^39^ ^]^ The analysis with the CircInteractome database showed multiple binding sites for cPKM and AGO2 (Figure S4C, Supporting Information). Upon reviewing the silver staining results, we found a differential band at ∼97 kDa in the cPKM probe group (black box). Western blot analyses and the RIP assay also confirmed the association between cPKM and AGO2 (Figure 4H,I). Based on the analyses performed using ENCORI (https://starbase.sysu.edu.cn/), CircMine (http://hpcc.siat.ac.cn/circmine), and Circ2GO (https://circ2GO.dkfz.de) databases, we predicted the potential target miRNAs of cPKM, and we identified two candidate miRNAs in the intersection of the three databases (Figure 4J, Table S4, Supporting Information). The RAP assays showed that miR‐199a‐5p but not miR‐1301‐3p was significantly enriched by the cPKM probe (Figure 4K). AGO2‐RIP assay showed that cPKM was significantly enriched after miR‐199a‐5p overexpression (Figure 4L). RNA‐FISH showed that cPKM and miR‐199a‐5p were co‐localized in the cytoplasm (Figure 4M). miRNAs often cause post‐transcriptional repression by pairing with sites in the mRNA 3ʹUTR.^[^ ^40^ ^]^ Surprisingly, using the RNA22 tool (https://cm.jefferson.edu/rna22/Interactive/), we found a binding site for miR‐199a‐5p and STMN1 mRNA 3ʹUTRs (Figure S4D, Supporting Information). To substantiate that miR‐199a‐5p can interact with cPKM and STMN1, we constructed luciferase reporter vectors containing wild‐type and mutated putative binding sites of cPKM or STMN1 3ʹUTR, respectively (Figure 4N). The luciferase reporter assay showed that transfection with miR‐199a‐5p mimics the significantly reduced luciferase activity of cPKM or STMN1 wild‐type reporters but not the mutated reporters (Figure 4O). To further confirm if cPKM regulates STMN1 expression by competitively binding with miR‐199a‐5p, we performed RNA pull‐down assays using biotin‐labeled miR‐199a‐5p mimics. We found that both cPKM and STMN1 were significantly enriched by miR‐199a‐5p (Figure 4P); however, STMN1 enrichment was reduced after cPKM overexpression (Figure 4Q). Moreover, miR‐199a‐5p mimics reduced STMN1 mRNA and protein levels in ICC cells, whereas cPKM overexpression restored them (Figure 4R,S). Furthermore, miR‐199a‐5p mimics mediated the inhibition of HCCC‐9810 microtumor sphere growth, and migration was abolished by cPKM‐Δ111–115 (Figure 4T,U). These results suggest that cPKM upregulated STMN1 expression by sponging miR‐199a‐5p, thus promoting ICC cell proliferation and migration.

### 2.5 cPKM Exerts Intracellular and Extracellular Effects Via IGF2BP2‐STMN1/TGFB1 and miR‐199a‐5p‐STMN1 Bi‐Axis

**Figure 5:**
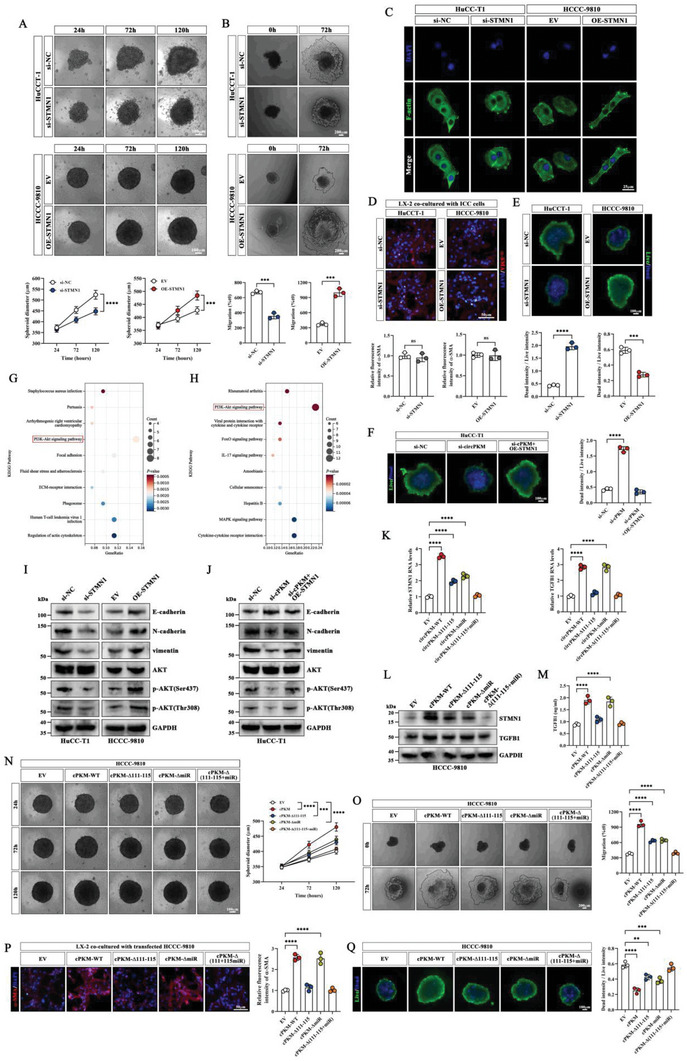
STMN1 regulates ICC malignant traits, and cPKM exerts intracellular and extracellular effects by regulating STMN1 and TGFB1 expression A) Representative images of KD‐STMN1 or OE‐STMN1 ICC microtumor spheroids and sphere diameters at different timepoints (*n* = 3). B) Representative images of KD‐STMN1 or OE‐STMN1 ICC microtumor spheroid migration and the relative migration area at different time points (*n* = 3). C) F‐actin (green) staining of the indicated ICC cells by phalloidin. D) Representative images of α‐SMA immunofluorescence and the relative fluorescence intensity in LX‐2 cells after co‐culture with KD‐STMN1 or OE‐STMN1 ICC cells (*n* = 3). E) Representative images of live (green)/dead (blue) cell double staining of KD‐STMN1 or OE‐STMN1 ICC microtumor spheroids after paclitaxel treatment for 48 h and the ratio of fluorescence intensity of dead cells to live cells (*n* = 3). F) Representative images of live (green)/dead (blue) cell double staining of the indicated ICC microtumor spheroids after paclitaxel treatment for 48 h and the ratio of fluorescence intensity of dead cells to live cells (*n* = 3). G) The bubble plot shows the top 10 enriched KEGG pathways in differentially expressed genes between STMN1 high‐ and low‐expression subgroups based on the TCGA CHOL RNA‐seq data. H) The bubble plot shows the top 10 enriched KEGG pathways after cPKM knockdown. I) Western blot analysis of E‐cadherin, N‐cadherin, vimentin, AKT, p‐AKT (Ser473), and p‐AKT (Thr308) in KD‐STMN1 or OE‐STMN1 ICC cells. J) Western blot analysis of E‐cadherin, N‐cadherin, vimentin, AKT, p‐AKT (Ser473), and p‐AKT (Thr308) in the indicated ICC cells. K,L) The mRNA and protein levels of STMN1 and TGFB1 in the indicated HCCC‐9810 cells were analyzed by K) qRT‐PCR and L) Western blot analysis (*n* = 3). M) The TGFB1 concentration in the supernatant of the indicated HCCC‐9810 cells was measured by ELISA (*n* = 3). N) Representative images of the indicated HCCC‐9810 microtumor spheroids and sphere diameters at different time points (*n* = 3). O) Representative images of the indicated HCCC‐9810 microtumor spheroid migration and the relative migration area at different time points (*n* = 3). P) Representative images of α‐SMA immunofluorescence and the relative fluorescence intensity in LX‐2 cells after co‐culture with the indicated HCCC‐9810 cells (*n* = 3). Q) Representative images of live (green)/dead (blue) cells double‐stained in the indicated HCCC‐9810 microtumor spheroids after paclitaxel treatment for 48 h and the ratio of fluorescence intensity of dead cells to live cells (*n* = 3). *n* indicates biological replicates. Data are the mean ± SD **p < 0.01, ***p < 0.001, ****p < 0.0001. Two‐way (A, N) or one‐way (F, K, M, O–Q) ANOVA, and unpaired two‐tailed Student's *t*‐tests (B, D, E).

Although STMN1 is closely associated with the progression of and poor prognosis in various tumors,^[^ ^21^, ^23^, ^41^, ^42^ ^]^ its role in ICC has not been reported. Analyses using the GEPIA2 (http://gepia2.cancer‐pku.cn/) database showed that STMN1 was significantly upregulated in cholangiocarcinoma tissues (Figure S5A, Supporting Information) and that patients with cholangiocarcinoma having a high expression level of STMN1 had poorer OS (Figure S5B, Supporting Information). We verified the expression level of STMN1 in ICC tissues using qRT‐PCR and found it to be highly expressed in most ICC tissues (Figure S5C, Supporting Information). Furthermore, survival analysis showed that high expression of STMN1 was associated with worse OS and RFS in patients with ICC (Figure S5D, Supporting Information). To explore the biological effects of STMN1 in ICC, we knocked down or overexpressed STMN1 in ICC cells and verified the efficiency of manipulated expression of STMN1 (Figure S5E, Supporting Information). Consequently, we found that the proliferation and migration of HuCC‐T1 microtumor spheroids were significantly inhibited with STMN1 knockdown and that the filamentous pseudopods were fewer; conversely, STMN1 overexpression promoted the proliferation and migration of HCCC‐9810 microtumor spheroids and formation of filamentous pseudopods (**Figure** **5**A–C). However, altered STMN1 expression in ICC cells did not affect myofibroblast activation in HSCs (Figure 5D). The expression levels of STMN1 were correlated with paclitaxel resistance.^[^ ^22^, ^23^ ^]^ We performed live/dead cell double staining of ICC microtumor spheroids under paclitaxel treatment and found that the resistance of ICC microtumor spheroids to paclitaxel was reduced after STMN1 knockdown but increased after STMN1 overexpression (Figure 5E). We further found that the reduced paclitaxel resistance of ICC microtumor spheroids caused by cPKM knockdown could be restored by STMN1 overexpression (Figure 5F).

To explore the potential mechanisms by which STMN1 promotes the malignant behaviors of ICC cells, we divided the cholangiocarcinoma patients in The Cancer Genome Atlas database into STMN1 high‐expression group (top 25%) and STMN1 low‐expression group (bottom 25%) according to their STMN1 expression levels. The Kyoto Encyclopedia of Genes and Genomes (KEGG) enrichment analysis of DEGs between the two subgroups showed that the PI3K/AKT pathway was significantly enriched (Figure 5G). Moreover, we found that the PI3K/AKT pathway was also significantly enriched after cPKM knockdown (Figure 5H). Using Western blot analysis, we found that both STMN1 knockdown and cPKM knockdown reduced the levels of phosphorylated AKT, whereas STMN1 overexpression promoted AKT phosphorylation and restored the effect of cPKM knockdown on AKT phosphorylation (Figure 5I,J). Moreover, N‐cadherin and vimentin showed a consistent trend in alteration of protein levels while E‐cadherin in contrast (Figure 5I,J).

To investigate whether cPKM exerts its biological functions in ICC cells by regulating IGF2BP2 and miR‐199a‐5p, we transfected wild‐type cPKM plasmids and cPKM plasmids with an IGF2BP2 binding site mutation (cPKM‐Δ111–115) or miR‐199a‐5p binding site mutation (cPKM‐ΔmiR) or both [cPKM‐Δ(111–115+miR)] into HCCC‐9810 cells, respectively. Compared with the empty vector, cPKM‐WT significantly increased the expression of STMN1 and TGFB1. Furthermore, both cPKM‐Δ111–115 and cPKM‐ΔmiR partially enhanced STMN1 expression. TGFB1 expression was only partially upregulated by cPKM‐ΔmiR. cPKM‐Δ(111–115+miR) did not affect STMN1 and TGFB1 expression (Figure 5K–M). This suggests that cPKM enhanced STMN1 expression by interacting with IGF2BP2 and miR‐199a‐5p, whereas TGFB1 was regulated by the interaction of cPKM with only IGF2BP2 and not miR‐199a‐5p. Furthermore, in vitro biological function assays showed that both cPKM‐Δ111–115 and cPKM‐ΔmiR partially promoted ICC tumorsphere proliferation, migration, and paclitaxel resistance. Notably, HSCs were only partially activated by cPKM‐ΔmiR, and cPKM‐Δ(111–115+miR) had almost no effect on ICC cells and HSCs (Figure 5N–Q). The above results indicate that cPKM promotes ICC tumor proliferation, migration, and paclitaxel resistance by upregulating STMN1 expression and activating the AKT pathway through interactions with IGF2BP2 and miR‐199a‐5p. Taken together, cPKM could enhance HSC myofibroblast activation by promoting TGFB1 secretion from ICC cells through interactions with IGF2BP2.

### 2.6 Preparation and Characterization of siRNA and PTX Co‐Loaded Nanosystem

**Figure 6:**
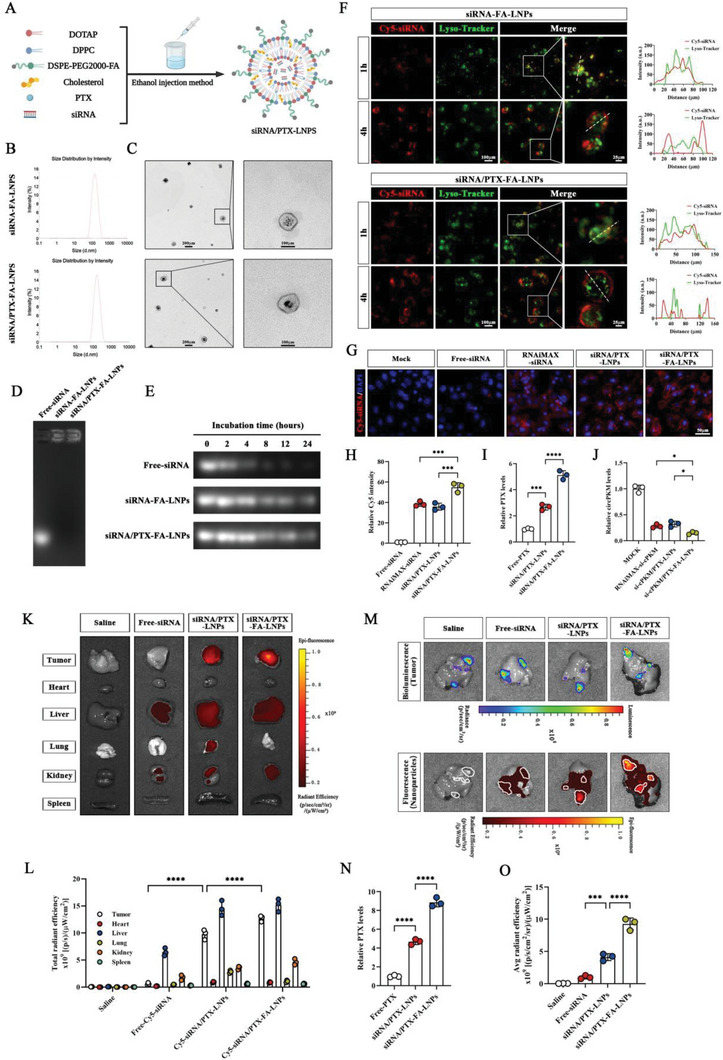
Preparation and characterization of siRNA and PTX co‐loaded nanosystems A) A schematic of the synthesis of siRNA and PTX co‐loaded nanosystems (created using BioRender.com). B) Size distribution of siRNA‐FA‐LNPs and siRNA/PTX‐FA‐LNPs as determined by DLS. C) Electron microscopy images of siRNA‐FA‐LNPs and siRNA/PTX‐FA‐LNPs. D) Electrophoretic mobility of free siRNA, siRNA‐FA‐LNPs, and siRNA/PTX‐FA‐LNPs. E) The serum stability of siRNAs in free siRNA, siRNA‐FA‐LNPs, and siRNA/PTX‐FA‐LNPs was visualized by agarose gel electrophoresis at different incubation durations. F) The cellular sublocalization of Cy5 (red) labeled siRNA‐FA‐LNPs or siRNA/PTX‐FA‐LNPs and lysosomes (green) at 1 h and 4 h. Cy5‐siRNA and LysoTracker signals are shown along the arrows. G) Images of Cy5‐siRNA in ICC cells after the indicated treatments. H) The relative fluorescence intensity of Cy5‐siRNA in ICC cells after the indicated treatments. I) The relative levels of PTX in ICC cells after the indicated treatments. J) The knockdown efficiency of cPKM in the indicated groups. K,L) Ex vivo fluorescence images and statistical analysis of tumors and major organs with the indicated treatments. N) The relative levels of PTX in subcutaneous xenografts after the indicated treatments. M,O) Ex vivo livers’ fluorescence and bioluminescence images and associated statistical analyses with the indicated treatments. Tumor sites were marked with white lines based on bioluminescence detection (*n* = 3). *n* indicates biological replicates (H–J) or individual mice (L, N, O). Data are the mean ± SD *p < 0.05, ***p < 0.001, ****p < 0.0001. One‐way (H‐J, N, O) or two‐way (L) ANOVA.

Since cPKM has an important biological effect on ICC, we developed siRNA and PTX co‐loaded nanosystems to explore potential therapies for ICC. First, the liposome components DOTAP, DPPC, DSPE‐PEG (2000)‐FA, and cholesterol (with a molar ratio of 50:10:38.5:1.5) were dissolved in ethanol, and for siRNA/PTX‐LNPs, PTX [5% (w/w) of total lipids)] was also added. Then, the lipid solution was mixed with the aqueous siRNA solution to form lipid nanoparticles (LNPs, **Figure** **6**A). The folate receptor (FR) is overexpressed in many human cancers and has become a useful target for tumor‐specific drug delivery.^[^ ^43^ ^]^ Analysis using the GEPIA2 database showed that FOLR1 (FR alpha) was significantly upregulated in cholangiocarcinoma tissues (Figure S6A, Supporting Information), and thus, we used folate‐conjugated DSPE‐PEG (2000) for targeting ICC cells. Dynamic light scattering (DLS) results showed that the hydrodynamic diameters of siRNA‐LNPs and siRNA/PTX‐LNPs were 138.6 (PDI: 0.112) and 152.2 nm (PDI: 0.098), respectively (Figure 6B). The sizes determined by transmission electron microscopy (TEM) were consistent with those measured by DLS (Figure 6C). The results of the agarose gel electrophoresis assay showed that siRNA‐LNPs did not show electrophoretic shift compared to naked siRNA (free RNA), indicating that siRNA was efficiently packaged into LNPs (Figure 6D). The encapsulation efficiency of siRNA was further determined to be 96% using RiboGreen assay. The encapsulation efficiency of PTX was 52%, which was determined by high‐performance liquid chromatography (HPLC).

Naked RNA is unstable in the plasma because it is rapidly degraded by nucleases.^[^ ^44^ ^]^ To assess the stability of siRNA in LNPs, we incubated free RNA, siRNA‐FA‐LNPs, and siRNA/PTX‐FA‐LNPs with 50% fetal bovine serum (FBS) at 37 °C for different durations. As shown in Figure 6E, siRNA‐FA‐LNPs and siRNA/PTX‐FA‐LNPs significantly improved the serum stability of siRNA when compared with free RNA. Moreover, we further investigated the endo/lysosomal escape ability of LNPs. To this end, siRNA‐FA‐LNPs or siRNA/PTX‐FA‐LNPs were incubated with ICC cells, and lysosomes were labeled using LysoTracker Green. We found that after 1 h of incubation, Cy5‐siRNA (red) was mostly located within the lysosomes (green); however, at 4 h, the green and red fluorescent signals were separated, indicating that the nanoparticles successfully escaped from the lysosomes (Figure 6F). The cellular uptake assay confirmed that siRNA/PTX‐LNPs could effectively deliver siRNA and PTX to ICC cells, and more importantly, it confirmed that siRNA/PTX‐FA‐LNPs had higher amounts of intracellular siRNA and PTX (Figure 6G–I). Additionally, siRNA/PTX‐FA‐LNPs exhibited the best cPKM‐silencing efficiency (Figure 6J).

Next, we assessed the in vivo biological distribution of LNPs in subcutaneous and orthotopic tumor (stably expressing luciferase)‐bearing mice. Fluorescence or bioluminescence imaging was performed 48 h after tail vein injection of saline, free siRNA, siRNA/PTX‐LNPs, or siRNA/PTX FA‐LNPs (ex vivo fluorescence imaging of tumors and other major organs for the subcutaneous xenograft model, ex vivo bioluminescence and fluorescence imaging of livers for the orthotopic tumor model). The results showed that LNP groups exhibited higher levels of fluorescence intensity in tumors compared to the free siRNA group and that folate could further increase the accumulation of nanoparticles in the tumor (Figure 6K–M,O). In addition, the highest PTX content was found in subcutaneous xenografts after the siRNA/PTX‐FA‐LNP treatment (Figure 6N). As nanoparticles are often taken up by resident macrophages in the liver, we used flow cytometry to further analyze the percentage of Cy5‐positive cells among Kupffer cells or tumor cells in the livers of orthotopic tumor model mice with different LNP treatments. We found that compared with the siRNA/PTX‐LNPs, the uptake of nanoparticles by tumor cells was increased, and that by Kupffer cells was decreased in the siRNA/PTX‐FA‐LNP group. In addition, in the siRNA/PTX‐FA‐LNP group, the percentage of Cy5‐positive tumor cells was significantly higher than that of Cy5‐positive Kupffer cells (Figure S6B, Supporting Information). This finding may be attributed to polyethylene glycol (PEG) and folate. The above results suggest that siRNA/PTX‐FA‐LNPs can reduce phagocytosis by macrophages and effectively target ICC cells via folate.

### 2.7 Antitumor Effects of the siRNA/PTX Co‐Loaded Nanoparticles

**Figure 7:**
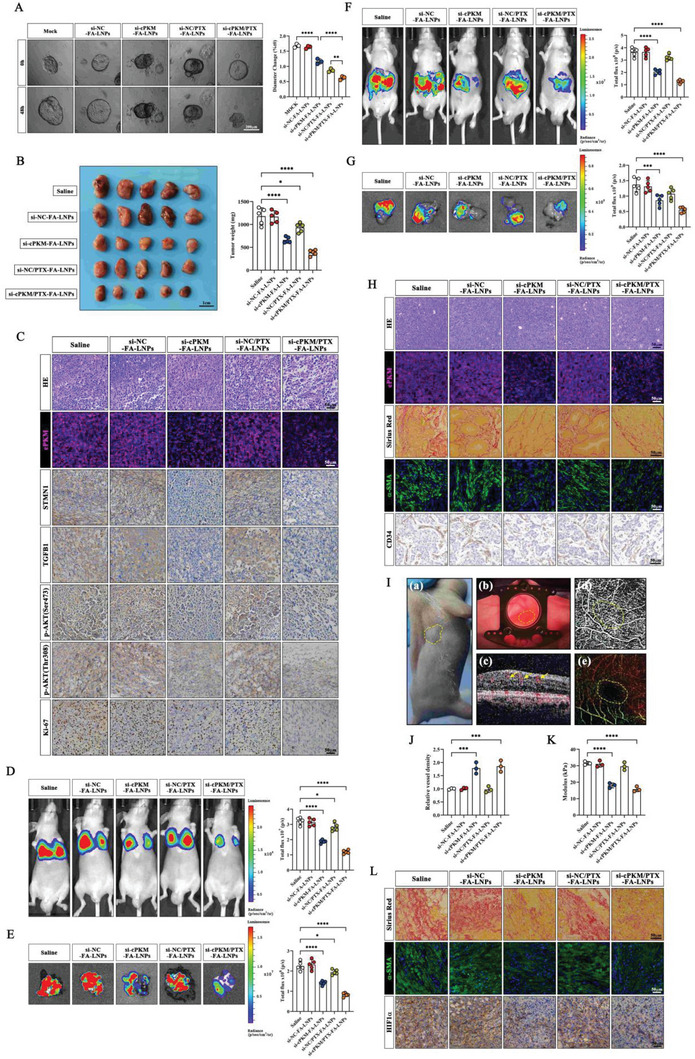
Antitumor effect of siRNA and PTX co‐loaded nanoparticles. A) Representative images and statistical analysis of ICC organoid growth after the indicated treatments (*n* = 3). B) Images and weights of subcutaneous xenografts after the indicated treatments (*n* = 5). C) Representative images of H&E staining, cPKM FISH staining, and immunohistochemistry staining for STMN1, TGFB1, p‐AKT (ser473), p‐AKT (Thr308), and Ki‐67 in the subcutaneous xenografts with the indicated treatments. D,E) Representative images and statistical analysis of D) in vivo and E) ex vivo lung bioluminescence in the lung metastasis model with the indicated treatments (*n* = 5). F,G) Representative images and statistical analysis of F) in vivo and G) ex vivo liver bioluminescence in the orthotopic tumor models with the indicated treatments (*n* = 5). H) Representative images of H&E staining, cPKM (FISH) staining, Sirius red staining, α‐SMA (IF) staining, and CD34 (IHC) staining in the orthotopic tumors with the indicated treatments. I) A schematic of microvascular OCT imaging. a) Subcutaneous tumor‐bearing nude mice. b) Images of tumor sites after dorsal skin window chamber (DSWC) mounting. c) Tissue cross‐section structural image (gray) and blood flow image (yellow arrow). d) A grayscale image of tumor microvasculature. e) The optical coherence tomography (OCT) image of tumor microvasculature. The yellow dotted line represents the tumor area. J) The relative tumor vascular intensity in the subcutaneous xenografts with the indicated treatments (*n* = 3). K) Young's modulus of the tumors with the indicated treatments (*n* = 3). L) Representative images of Sirius red staining, α‐SMA (IF), staining, and HIF1α (IHC) staining in the subcutaneous xenografts with the indicated treatments (*n* = 3). *n* indicates biological replicates (A) or individual mice (B, D–G, J, K). Data are the mean ± SD *p < 0.05, **p < 0.01, ***p < 0.001, ****p < 0.0001. One‐way ANOVA (A, B, D–G, J, K).

To explore the potential therapeutic effects of siRNA/PTX co‐loaded nanoparticles on ICC tumors, we constructed an ICC organoid. We found that si‐cPKM‐FA‐LNPs inhibited the growth of the organoid and that the efficacy of si‐NC/PTX‐FA‐LNPs was slightly better than that of free PTX treatment. The si‐cPKM/PTX‐FA‐LNP treatment exhibited a more pronounced inhibitory effect (**Figure** **7**A, Figure S6C, Supporting Information). To further validate the antitumor effects of siRNA/PTX‐FA‐LNPs, in vivo ICC tumor models in mice were established using HCCC‐9810 cells stably overexpressing cPKM. The results for the subcutaneous xenograft model were similar to those for the organoid model (Figure 7B, Figure S6D, Supporting Information). The RNA‐FISH assay confirmed that cPKM in xenografts was effectively silenced by si‐cPKM‐FA‐LNPs and si‐cPKM/PTX‐FA‐LNPs. IHC staining showed that si‐cPKM‐FA‐LNPs and si‐cPKM/PTX‐FA‐LNPs could significantly reduce STMN1 and TGFB1 expression and AKT phosphorylation. Moreover, compared with the control group, the number of Ki‐67‐positive cells was reduced in the si‐cPKM‐FA‐LNP group, and an even more significant reduction was observed in the si‐cPKM/PTX‐FA‐LNP group (Figure 7C). Next, we evaluated the biosafety of LNPs. There was no obvious difference in body weight among the different groups of mice (Figure S6E, Supporting Information), and no histopathological abnormalities were observed in major organs, including the heart, liver, spleen, lung, and kidney (Figure S6F, Supporting Information). We did not observe any significant changes in blood biochemical parameters, such as ALT, AST, BUN, and CREA (Figure S6G–J, Supporting Information). These results suggest that nanoparticles have no significant adverse effects and can be used as a safe agent for further in vivo applications.

In the lung metastasis model and orthotopic model, we observed similar results as we did in the subcutaneous xenograft model, with the si‐cPKM/PTX‐FA‐LNP group showing the best antitumor efficacy (Figure 7D–G). To exclude the possibility that the efficacy of LNPs in the orthotopic model was influenced by cPKM silencing in a portion of intrahepatic macrophages because of the uptake of nanoparticles, we estimated the level of cPKM in Kupffer cells using qRT‐PCR and found it to be significantly lower in Kupffer cells than in ICC cells (Figure S6K, Supporting Information). In addition, the growth and migration of HCCC‐9810 microtumor spheroids did not change after co‐culture with KD‐cPKM Kupffer cells (Figure S6L,M, Supporting Information). This suggests that the efficacy of LNPs has little to do with intrahepatic macrophages. In addition, we found that si‐cPKM‐FA‐LNPs and si‐cPKM/PTX‐FA‐LNPs reduced the degree of fibrosis and α‐SMA abundance and caused more microvessel openings in the orthotopic model (Figure 7H). The subcutaneous xenograft model with co‐implantation of HSCs and OE‐cPKM HCCC‐9810 cells was established to further verify if LNPs inhibited myofibroblast activation of HSCs by delivering si‐cPKM, thereby reducing fibrosis and causing collapsed microvessel opening in ICC tumors. The proportions of tumor cells, HSCs, and other cells in the xenografts of the co‐implantation model were determined by flow cytometry, which confirmed the successful construction of the model (tumor cells account for 57.98%, and HSCs account for 32.90%, Figure S6N, Supporting Information). We installed a dorsal skin window chamber on subcutaneous tumor‐bearing nude mice for optical coherence tomography imaging (Figure 7I). The results indicated that the tumor microvessel density was increased in si‐cPKM‐FA‐LNP and si‐cPKM/PTX‐FA‐LNP groups (Figure 7J). The abnormal tumor mechanical microenvironment, including dense and rigid extracellular matrix (ECM), high tumor stiffness, and solid stress, is a formidable physical barrier that shields cancer cells from therapeutic agents.^[^ ^45^ ^]^ To assess tumor stiffness, we examined Young's modulus of tumor tissue sections using atomic force microscopy. The results showed that Young's modulus of tumor tissue sections was lower in si‐cPKM‐FA‐LNP and si‐cPKM/PTX‐FA‐LNP groups than in the saline group (Figure 7K). In addition, less fibrosis and α‐SMA abundance and more microvessel opening were observed in si‐cPKM‐FA‐LNP and si‐cPKM/PTX‐FA‐LNP groups, which was consistent with the results for the orthotopic model (Figure 7L). Moreover, reduced HIF1α levels in si‐cPKM‐FA‐LNP and si‐cPKM/PTX‐FA‐LNP groups indicated that the opening of the collapsed vessels improved blood perfusion and reduced intratumoral hypoxia (Figure 7L). Collectively, these results suggest that siRNA/PTX co‐loaded nanoparticles have superior antitumor effects in multiple ICC tumor models.

### 2.8 The siRNA/PTX Co‐Loaded Nanoparticles Enhance the Efficacy of Standard Chemotherapy for ICC

**Figure 8:**
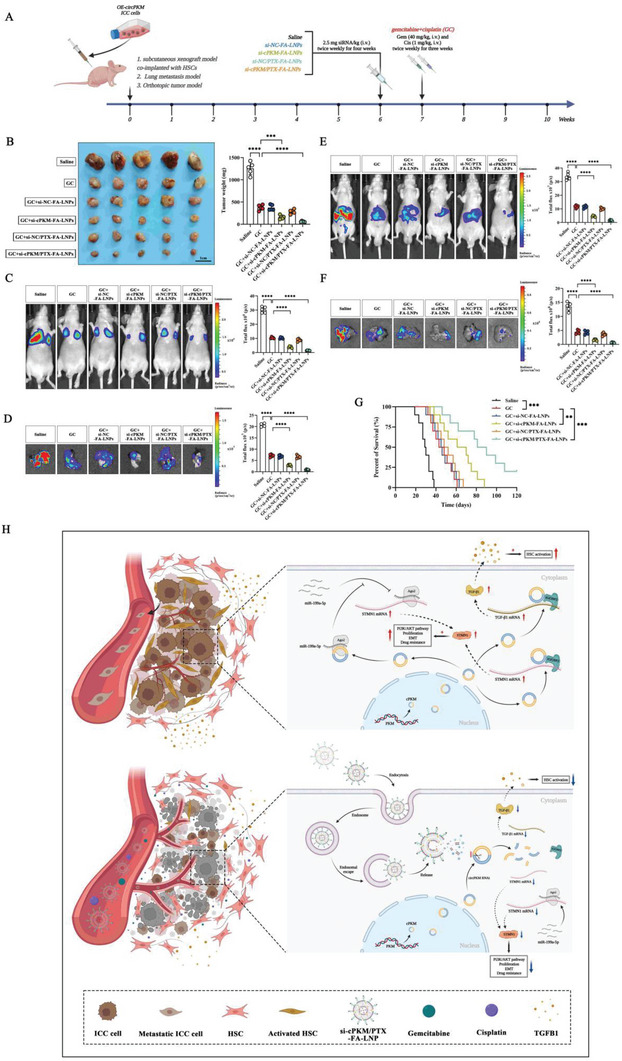
siRNA and paclitaxel co‐loaded nanoparticles enhance the efficacy of standard chemotherapy regimens. A) A schematic of the treatment regimen for ICC tumor models (created using BioRender.com). B) Images and weights of subcutaneous xenografts after the indicated treatment (*n* = 5). C,D) Representative images and statistical analysis of C) in vivo and ex vivo D) lung bioluminescence in the lung metastasis model with the indicated treatments (*n* = 5). E,F) Representative images and statistical analysis of in vivo E) and ex vivo F) liver bioluminescence in the orthotopic tumor models with the indicated treatments (*n* = 5). G) Survival curves of orthotopic tumor‐bearing mice after the indicated treatments (*n* = 10). H) Graphical illustration of nanotherapy targeting the cPKM‐STMN1/TGFB1 axis (created using BioRender.com). Lipid nanoparticles efficiently deliver siRNA and paclitaxel into ICC cells. siRNA‐mediated silencing of cPKM in ICC cells inhibits the expression of STMN1, weakens the proliferation and metastasis ability of ICC cells, and sensitizes them to paclitaxel. In addition, the inhibition of TGFB1 release reduces tumor stromal fibrosis, opens collapsed tumor microvessels, and enhances the efficacy of the standard chemotherapy regimen. *n* indicates individual mice. Data are the mean ± SD **p < 0.01, ***p < 0.001, ***p < 0.0001. One‐way ANOVA (B–F) and log‐rank test (G).

The above results demonstrate that the progression of ICC was inhibited to a certain extent after nanoparticle treatment, and more importantly, the fibrosis of ICC tumor stroma was reduced and microvessels were opened, collectively paving the way for efficient drug delivery. As the standard treatment practice for ICC is systemic chemotherapy with gemcitabine/cisplatin (GC),^[^ ^6^ ^]^ we wondered whether siRNA/PTX co‐loaded nanoparticles could enhance the therapeutic effect of the GC treatment. We compared the efficacy of GC alone and in combination with different treatments in mouse ICC tumor models (**Figure** **8**A). In subcutaneous xenograft models co‐implanted with HSCs and OE‐cPKM ICC cells, si‐cPKM‐FA‐LNPs enhanced the efficacy of GC. Compared to the GC monotherapy group, the combination of GC with si‐NC/PTX‐FA‐LNPs showed a modest increase in efficacy, but it was still better than the efficacy of GC combined with free PTX. The combination of GC and si‐cPKM/PTX‐FA‐LNPs exhibited the most potent antitumor effect (Figure 8B, Figure S6O, Supporting Information). In the lung metastasis model and orthotopic model of OE‐cPKM ICC cells, GC combined with si‐cPKM/PTX‐FA‐LNPs also demonstrated the most significant antitumor response (Figure 8C–F). In addition, the si‐cPKM/PTX‐FA‐LNP group showed the longest OS in the orthotopic model (Figure 8G). Overall, the siRNA/PTX co‐loaded nanoparticles were found to suppress ICC progression and improve the efficacy of standard chemotherapy regimens in multiple ICC tumor models.

## 3 Discussion

Notably, ICC is characterized by highly malignant growth and an abundant tumor stroma involving multiple molecular pathways and multiple cellular interactions.^[^ ^10^ ^]^ These disease features are implicated in its low survival rate and poor treatment outcome. With advances in high‐throughput RNA detection technologies and circRNA‐specific bioinformatics algorithms, numerous circRNAs have been identified in eukaryotes, and many have been shown to play important roles in the progression of tumors.^[^ ^24^, ^25^, ^46^ ^]^ However, the vast majority of current research on circRNAs has focused only on molecular mechanisms within tumor cells or signaling crosstalk in the tumor microenvironment. Few circRNAs that can affect both intracellular and extracellular factors have been reported. By performing circRNA microarray analysis of ICC tumor tissues and paired normal tissues in combination with public datasets, we report that cPKM, a circRNA, is upregulated in ICC. cPKM acts as a critical bridge that drives IGF2BP2 to bind to STMN1/TGFB1 mRNA, thus stabilizing TGFB1 and STMN1 mRNAs. cPKM also acts as a sponge for miR‐199a‐5p to upregulate STMN1 expression. By regulating the expression of TGFB1 and STMN1, cPKM exerts dual biological effects both intracellularly and extracellularly; it enhances the malignant characteristics of ICC cells themselves, activates HSCs, and promotes extracellular matrix fiber proliferation (Figure 8H).

TGFB1 is a key molecule in the activation and switching of HSCs to a myofibroblast‐like phenotype.^[^ ^14^, ^34^, ^35^ ^]^ The TGFB1 secreted by tumor cells in the hepatic microenvironment induces the activation of HSCs to produce many extracellular matrix components, such as collagen 1 and fibronectin, promoting dense matrix formation and shaping the microenvironment for tumor progression.^[^ ^10^, ^14^ ^]^ Few studies have investigated the factors regulating the level of TGFB1 secreted by tumor cells, and to our knowledge, the role of circRNAs in regulating progress has not been reported in ICC. In the present study, we found that ICC cells with high expression of cPKM secreted more TGFB1 and promoted greater activation of HSCs. Notably, in an orthotopic tumor model, we observed that high expression of cPKM promoted ICC stromal fiber proliferation and abnormal vascular collapse, which greatly resembles the tumor microenvironment characteristics of patients with ICC.

STMN1, also known as oncoprotein 18 (OP18), is a cytoplasmic phosphoprotein that regulates microtubule dynamics by promoting microtubule instability. Furthermore, it is involved in regulating the dynamic homeostasis of microtubules within cells, affecting cell morphology, motility, and division. High STMN1 levels have been detected in various malignancies, and they are associated with accelerated tumor progression.^[^ ^21^, ^23^, ^41^, ^42^ ^]^ In addition, STMN1 is an important response factor to clinical chemotherapy in patients with paclitaxel‐resistant tumors and is closely associated with chemoresistance of tumors.^[^ ^22^, ^23^ ^]^ However, to our knowledge, the expression level and the role of such an important cancer‐related protein in intrahepatic cholangiocarcinoma have not been reported so far. In the present study, we found for the first time that STMN1 is aberrantly highly expressed in ICC via regulation by cPKM, which in turn promotes ICC cell proliferation, metastasis, and paclitaxel resistance. Previous studies have shown that STMN1 can act as a signature molecule of the PI3K/AKT pathway in breast cancer and pelvic serous carcinomas.^[^ ^47^, ^48^ ^]^ We confirmed using bioinformatics analysis and experiments that STMN1 is also important for activating the PI3K/AKT pathway in ICC.

CircRNAs have great potential as novel therapeutic targets for cancer therapy.^[^ ^17^, ^49^ ^]^ For example, Rossi et al. found that inhibiting circZNF609 increased the sensitivity of multiple cancer cells to microtubule‐targeted chemotherapeutic agents^[^ ^50^ ^]^; Du et al. found that exogenous overexpression of cNFIB enhanced the antitumor effects of trametinib^[^ ^18^ ^]^; and in one of our previous studies, we also showed that silencing circ‐MTHFD1L followed by co‐administration of olaparib increased the sensitivity of pancreatic cancer to gemcitabine.^[^ ^51^ ^]^ However, interfering with circRNAs at tumor sites in a controlled and precise manner is still a difficult task. In recent times, nanomedicines have made a splash in clinical treatment, and some studies based on nanotechnology have attempted tumor treatment by interfering with circRNAs. For instance, Du et al. effectively inhibited the proliferation and metastasis of HCC in vivo by synthesizing PAE‐siRNA nanoparticles targeting circMDK.^[^ ^52^ ^]^ In addition, Li et al. encapsulated antigen‐encoding circRNA in liposomes and showed superior antitumor efficacy in various mouse tumor models by triggering innate and adaptive immune activation.^[^ ^53^ ^]^

In this study, considering the characteristics of ICC and the discovery of circRNA with intracellular and extracellular effects, we explored a Trojan horse nanotherapy strategy. First, we synthesized a folate‐conjugated nanosystem to act as a Trojan horse by loading si‐cPKM and paclitaxel, the two “pioneer soldiers.” Taking advantage of the high penetration and targeting properties of nanoparticles, the nanosystem brought them safely and effectively into the solid ICC tumor like the city of Troy. Then, using ICC organoid models and multiple mouse ICC tumor models, we demonstrated that si‐cPKM could not only kill ICC internally in combination with paclitaxel but also reduce ICC tumor stromal fibrosis and increase microvascular opening, thus opening the gate for drug treatment. Finally, the external main force (GC) can flood into ICC through the open gates, thus achieving significant therapeutic effects and improving the survival of ICC mouse models. This strategy is the first to achieve effective sensitization of tumors to paclitaxel through a nanosystem with co‐loading of siRNA and paclitaxel in ICC; it is also the first to achieve effective treatment of ICC through internal and external synergistic potentiation.

In summary, our findings reveal that cPKM enhances ICC growth, metastasis, and resistance to paclitaxel by promoting STMN1 and TGFB1 expression and by causing abnormal activation of HSCs, exacerbating fibrosis, and ultimately leading to vascular collapse. The Trojan horse nanotherapy targeting the cPKM–STMN1/TGFB1 axis is a promising new strategy for ICC treatment.

## 4 Experimental Section

### Patients and Specimens

Specimens of intrahepatic bile duct cancers and adjacent normal tissues were collected from 95 patients in the Department of Hepatobiliary Surgery, Fujian Provincial Hospital between January 2016 and June 2019. Among them, five pairs were used for circRNA microarray analysis and the remaining 90 pairs were used for validation. Detailed clinicopathological data, such as sex, age, tumor size, and TNM stage, were obtained from hospital records. Patients or guardians signed an informed consent form before participation in this study.

### Cell Cultures

Human normal intrahepatic bile duct cells (HIBEpiC) and mouse liver Kupffer cells were purchased from Procell Life Science and Technology (Wuhan, China). HEK‐293T cells, the hepatic stellate cell line LX‐2, and the ICC cell lines HuCC‐T1, HCCC‐9810, and RBE were purchased from Cellcook Biotech (Guangzhou, China). HIBEpiC cells were cultured in a complete culture medium of human intrahepatic bile duct epithelial cells (Procell Life Science and Technology); mouse liver Kupffer cells were cultured in a complete culture medium of mouse liver Kupffer cells (Procell Life Science and Technology); HEK‐293T cells and LX‐2 cells were cultured in DMEM (Gibco, CA, USA); HuCC‐T1 cells, HCCC‐9810 cells, and RBE cells were cultured in RPMI‐1640 medium (Gibco, CA, USA). The media for all the cell lines contained 10% FBS (Gibco, CA, USA), 100 U mL^−1^ penicillin, and 100 µg mL^−1^ streptomycin (Gibco, CA, USA), and cells were cultured in a humidified incubator with 5% CO_2_ at 37 °C.

### CircRNA Microarray Analysis

Sample preparation and microarray hybridization were done according to Arraystar's standard protocols. Briefly, total RNAs were digested with RNase R (Epicentre, USA) to remove linear RNAs and enrich circular RNAs. Then, the enriched circular RNAs were amplified and transcribed into fluorescent cRNA using a random priming method (Arraystar Super RNA Labeling Kit; Arraystar). The labeled cRNAs were hybridized onto Arraystar Human circRNA Array V2 (8×15K, Arraystar). After washing the slides, the arrays were scanned using Agilent Scanner G2505C. Agilent Feature Extraction software (version 11.0.1.1) was used to analyze acquired array images.

### Microarray Data Download and Pre‐Processing

The ICC dataset GSE181523 was obtained from the GEO database (http://wwwncbinlmnihgov/geo). The data platform of GSE39454 was GPL21825, which was consistent with the circRNA microarray data. The probes were converted into the corresponding circRNA ID according to the annotation information of platforms. Batch effects between GSE3112 and the own microarray data were eliminated using the ComBat algorithm in the “SVA” R package, and the two datasets were merged for subsequent analysis.

### RNA Sequencing

RNA sequencing was performed by Seqhealth Technology (Wuhan, China). Briefly, total RNAs were extracted from ICC cells using the TRIzol reagent (Invitrogen, CA, USA). Total RNAs (2 µg) were used for stranded RNA sequencing library preparation using KC‐Digital Stranded mRNA Library Prep Kit for Illumina (Seqhealth Technology, Wuhan, China) according to the manufacturer's instructions. The kit eliminated duplication bias in PCR and sequencing steps using a unique molecular identifier of eight random bases to label the pre‐amplified cDNA molecules. The library products corresponding to 200–500 bp were enriched, quantified, and finally sequenced on the DNBSEQ‐T7 sequencer (MGI Tech, Shenzhen, China) with the PE150 model.

### Screening for Differentially Expressed Genes and Enrichment Analysis

The “limma” R package was used to infilter differentially expressed genes (DEGs). Gene ontology functional annotation and the KEGG pathway analysis of DEGs were conducted using the “clusterProfiler” R package.

### Weighted Gene Co‐Expression Network Analysis (WGCNA)

The “WGCNA” R package was used to construct weighted co‐expression networks. First, the “goodsamplesgenes” function was used to identify missing values and collate the datasets. Then, according to the criteria of the scale‐free network, the appropriate soft threshold *β* (range, 1–30) was selected using the “pickSoftThreshold” function in the WGCNA package. The disordered neighborhood relationships between genes were truncated based on the soft threshold size, followed by transforming the matrix data into a neighborhood matrix and building a scale‐free topological network. The adjacency matrix was transformed into a topological overlap matrix on the basis of the topological overlap differences in network connection strength. Subsequently, a hierarchical clustering dendrogram was further constructed, and gene clustering and dynamic module identification were performed. Finally, the correlations of module eigengenes with clinical features were calculated, and then, the heatmaps of module‐clinical trait correlations based on module eigengenes were plotted. GS and MM were also calculated to assess the significance of genes and estimate the correlation between genes and modules. In this study, the soft threshold *β* was 20. The other parameters were as follows: networkType = “signed”, minModuleSize = 30, mergeCutHeight = 0.25, and deepSplit = 2.

### SiRNA, Mimics, Plasmid Synthesis, and Lentivirus Infection

SiRNAs and mimics were synthesized by Shangya (Fuzhou, China) and transfected into ICC cells using RNAiMAX (Invitrogen, CA, USA) according to the manufacturer's instructions. The plasmid and the packaged lentivirus were purchased from Genechem (Shanghai, China) and used to construct stably transfected cell lines according to the manufacturer's instructions, which was followed by puromycin selection for four weeks.

### RNA Extraction and Quantitative Real‐Time PCR (qRT‐PCR) Analysis

According to the manufacturer's protocol, total RNA from ICC tissues or cell lines was isolated using the TRIzol reagent (Invitrogen, CA, USA). Reverse transcription was performed using PrimeScript RT Reagent Kit (Takara, Dalian, China). Bulge‐loop miRNA RT‐qPCR primers were applied to determine the level of miRNAs. Real‐time PCR reactions were performed using StepOnePlus Real‐Time PCR System (Thermo Fisher Scientific, MA, USA). The program settings on temperature cycling were followed according to the manufacturer's instructions. The relative circRNA/mRNA and miRNA expression levels were normalized to GAPDH and U6, respectively, using the 2^−ΔΔCT^ method.

### Ribonuclease R (RNase R) Treatment

Total RNA was incubated for 30 min at 37 °C with or without 3 U µg^−1^ RNase R (Geenseed, Guangzhou, China) according to the manufacturer's instructions, and then, the expression levels of cPKM and PKM mRNAs were quantified by qRT‐PCR.

### Actinomycin D Assay

Cells were treated with 2 µg mL^−1^ actinomycin D (Sigma‐Aldrich, MO, USA) and were harvested at different treatment timepoints. The RNA expression levels were quantified by qRT‐PCR.

### Immunohistochemistry (IHC) and Sirius Red Staining

Tumor tissues were fixed in 4% paraformaldehyde and embedded in paraffin. Tissue sections were deparaffinized and rehydrated, which was followed by antigen retrieval through heat mediation in citrate buffer. Samples were blocked with 5% BSA for 1 h. Primary antibodies were incubated overnight at 4 °C, which was followed by incubation with secondary antibodies at room temperature for 1 h. Diaminobenzidine (DAB) solution was used for chromogenic reaction. For Sirius red staining, tissue sections were stained with 0.1% Sirius red dissolved in saturated picric acid.

### Immunofluorescence (IF)

Samples were fixed in 4% paraformaldehyde at room temperature for 15 min, permeabilized with 0.2% Triton X‐100 for 10 min, and then blocked with 5% BSA at room temperature for 1 h. The slices were incubated overnight with primary antibodies at 4 °C and then incubated with fluorescent secondary antibodies at room temperature for 1 h. Samples were mounted after staining with diamidino‐2‐phenylindole (DAPI) dye.

### Fluorescent In Situ Hybridization (FISH)

Specific fluorescently labeled cPKM and miR‐199a‐5p probes were designed and synthesized by Servicebio (Wuhan, China). After fixation, permeabilization, and prehybridization, the samples were hybridized overnight with the probes in a hybridization buffer at 37 °C. The hybridization buffer was then gradually washed off with 4× SSC (including 0.1% Tween‐20), 2× SSC, and 1× SSC at 42 °C. Nuclei were counterstained with DAPI.

### Western Blot Analysis

In brief, proteins were isolated from ICC cells and tumor tissues using radioimmunoprecipitation assay (RIPA) buffer (Solarbio, Beijing, China) supplemented with proteinase inhibitors and a phosphatase inhibitor. The protein concentration was determined with a bicinchoninic acid reagent (Beyotime, Beijing, China). Proteins were separated using sodium dodecyl sulfate‐polyacrylamide gel electrophoresis and then transferred onto polyvinylidene difluoride membranes (Merck Millipore, MA, USA). After the membranes were blocked in 5% skim milk for 1 h, they were incubated with primary antibodies overnight at 4 °C. Next, the membranes were incubated with secondary antibodies at room temperature for 1 h. The targeted proteins were detected using the Pierce ECL Western Blotting kit (Thermo Fisher Scientific, MA, US) with ChemiDoc MP Imaging System (Bio‐Rad, CA, USA). GAPDH was used as the loading control in this study.

### Agarose Gel Electrophoresis

Nucleic acid samples were loaded on 2% (w/v) agarose gel and then separated using electrophoresis in Tris‐acetate‐EDTA running buffer at 120 V for 30 min. Gel images were visualized by ChemiDoc MP Imaging System (Bio‐Rad, CA, USA).

### Microtumor Spheroids Formation and Growth Assay

Tumor cell monolayers were washed twice with PBS, and then, cell dissociation enzymes were added to obtain single‐cell suspensions without cell clusters. Cells were counted using a hemocytometer, and the cell suspension was diluted according to the optimal cell density for each cell line to obtain 0.5–2 × 10^4^ cells mL^−1^. Then, using a multichannel pipette, cell suspension (200 µL per well) was dispensed into ultra‐low attachment (ULA) 96‐well round‐bottom plates (Corning, NY, USA). Next, the plates were transferred to an incubator (37 °C, 5% CO_2_, 95% humidity). Finally, images of microtumor spheroids were acquired after culturing for different durations and the diameter was calculated.

### Microtumor Spheroid‐Based Migration Assays

Herein, 200 mL per well of culture medium supplemented with 2% FBS was distributed into flat‐bottom 96‐well plates (Corning, NY, USA) coated with 0.1% (v/v) gelatin (Sigma‐Aldrich, MO, USA). Formation of microtumor spheroids was visually confirmed before the migration assay. Microtumor spheroids were transferred in a volume of 100 mL into each well of the migration plate, resulting in a final volume of 300 mL per well. Images were obtained at 0 h and 72 h after microtumor spheroid adhesion, and the area covered by migrating cells was quantified by Image J software.

### Enzyme‐Linked Immunosorbent (ELISA)

Cell culture supernatants were collected and centrifuged at 1000 *g* for 10 min. The secretion level of TGFB1 in supernatants was detected using a Human TGFB1 ELISA kit according to the manufacturer's protocol (Enzyme‐linked Biotechnology, Shanghai, China).

### Co‐Culture Assay

For the co‐culture model of ICC cells and HSCs, six‐well Transwell chambers with 0.4‐µm porous polycarbonate membranes (Corning Incorporated Life Sciences) were used. ICC cells were seeded in the upper chamber and cultured in RPMI‐1640 medium containing 10% FBS. LX‐2 cells were seeded in the lower chamber and cultured in DMEM containing 10% FBS. The cells were co‐cultured for 48 h and then used for subsequent experiments. For the co‐culture model of ICC tumor spheroids and Kupffer cells, 96‐well Transwell chambers with 0.4‐µm porous polycarbonate membranes (Corning Incorporated Life Sciences) were used. ICC tumor spheroids were placed in the lower chamber and cultured in RPMI‐1640 medium containing 10% FBS. Kupffer cells were seeded in the upper chamber and cultured in a complete culture medium of mouse liver Kupffer cells.

### Live/Dead Cell Double Staining Assay

After microtumor spheroid formation, they were treated with PTX (0.5 µM) for 48 h. Subsequently, cells were stained with a live (Calcein‐AM)/dead (NucBlue D) staining kit (BestBio, Shanghai, China) according to the manufacturer's instructions, and images were obtained using a fluorescence microscope (Leica, Wetzlar, Germany).

### RNA Antisense Purification (RAP) Assays

The RAP assay was performed using a BersinBio RNA antisense purification kit (BersinBio, Guangzhou, China) following the manufacturer's protocol. The coprecipitated proteins were detected using Silver Stain Kit (Solarbio, Beijing, China) and Western blot analysis. The coprecipitated RNA was detected using qRT‐PCR.

### RNA Immunoprecipitation (RIP) Assays

RIP assay was performed using a Magna RIP RNA‐binding protein immunoprecipitation kit (Merck Millipore, MA, USA) according to the manufacturer's protocol. The coprecipitated RNA was detected using qRT‐PCR.

### Methylated RNA Immunoprecipitation (MeRIP) Assays

The MeRIP assay was performed using Magna MeRIP m6A Kit (Merck Millipore, MA, USA) following the manufacturer's protocol. The coprecipitated RNA was detected using qRT‐PCR.

### Luciferase Reporter Assay

The wild‐type and mutant plasmids of STMN1 3ʹ UTR binding cPKM or miR‐199a‐5p were cloned into PGL3 luciferase plasmids (Genechem, Shanghai, China) and co‐transfected with cPKM plasmids or miR‐199a‐5p mimics; the wild‐type and mutant plasmids of TGFB1 3ʹ UTR binding cPKM were cloned into PGL3 luciferase plasmids and co‐transfected with cPKM plasmids; the wild‐type and mutant plasmids of cPKM binding miR‐199a‐5p were cloned into PGL3 luciferase plasmids and co‐transfected with miR‐199a‐5p mimics into 293T cells. At 48 h after transfection, the luciferase activities were detected using the dual‐luciferase reporter assay system (Promega, WI, USA) according to the manufacturer's protocol. The luciferase activities were normalized to the corresponding Renilla luciferase activities.

### Preparation and Characterization of Lipid Nanoparticles (LNPs)

Herein, DOTAP, DPPC, DSPE‐PEG (2000)/DSPE‐PEG (2000)‐FA, and cholesterol (with molar ratio of 50:10:38.5:1.5) were dissolved in ethanol to obtain a lipid mixture. For siRNA/PTX‐LNPs, PTX [5% (w/w) of total lipids] was also added. The lipid mixture (10 mg mL^−1^) was mixed with 20‐µM siRNA in a 1:2 ratio (v/v) through ice bath ultrasound sonication. The 30‐kDa molecular weight cutoff (MWCO) membrane was used to concentrate the LNPs by ultrafiltration and then washed twice with PBS. DLS and polydispersity index (PDI) of LNPs were characterized by Zeta Sizer (NanoZS, Malvern, U.K.). The morphology was imaged by TEM (Hitachi, Tokyo, Japan). The encapsulation efficiency of siRNA in LNPs was determined by measuring the fluorescence intensity of crude LNPs before ultrafiltration with the RiboGreen kit (Invitrogen, CA, USA). siRNA encapsulation efficiency = (F1‐F2)/F1 × 100%, where F1 and F2 are fluorescence intensities with and without Triton X‐100, respectively. The encapsulation efficiency of PTX in LNPs was measured by high‐performance liquid chromatography (HPLC, Agilent Technologies, CA, USA). The chromatographic conditions were as follows: mobile phase, acetonitrile, and water (60:40, v/v); detection wavelength, 227 nm; column temperature, 25 °C; and flow rate, 0.5 mL mi^−1^n. PTX encapsulation efficiency = *W*_1_/*W*_2_ × 100%, where *W*_1_ and *W*_2_ are the weights of PTX in LNPs and input, respectively.

### Cellular uptake of LNPs

To determine the cellular uptake of siRNA, the cells were incubated with free‐Cy5‐siRNA, RNAiMAX‐Cy5‐siRNA, or Cy5‐siRNA/PTX(‐FA)‐LNPs (with an amount equal to that of free‐Cy5‐siRNA). The cells were lysed with RIPA lysis buffer, and the fluorescence intensity of Cy5 was determined using a SpectraMax iD5 microplate reader (Molecular Devices, CA, USA). To determine the cellular uptake of PTX, cells were incubated with free PTX or siRNA/PTX(‐FA)‐LNPs (with an amount equal to that of free PTX). The cells were lysed with RIPA lysis buffer, and the concentration of PTX was determined by HPLC. To determine intracellular delivery, the cells were incubated with Cy5‐siRNA‐FA‐LNPs or Cy5‐siRNA/PTX‐FA‐LNPs. At predetermined time intervals, the cells were washed with PBS, counterstained with LysoTracker Green (Solarbio, Beijing, China) for 30 min, and then observed using a confocal microscope (Leica, Wetzlar, Germany).

### Organoid Culture

Organoids were derived from the tumor tissues of patients with ICC. Tumor tissues were cut into small pieces and digested with MasterAim tissue enzymatic solution I (StemCell Technologies, Vancouver, Canada) and advanced DMEM/F12 (Gibco, CA, USA) for 1 h and then further digested with MasterAim tissue enzymatic solution II (StemCell Technologies, Vancouver, Canada). Digestion was stopped by adding advanced DMEM/F12 containing 10% FBS. Cells were then resuspended in Matrigel (Corning, NY, USA) and plated into 24‐well plates at 37 °C for 30 min to allow solidification. The cholangiocarcinoma organoid was supplemented with a complete medium (BioGenous, Hangzhou, China) for organoid growth.

### Animal Experiments

Male Balb/c nude mice (4–6 weeks old) were purchased from Shanghai SLAC laboratory Animal Co., Ltd. (Shanghai, China).

### Biodistribution of LNPs

Balb/c nude mice with a subcutaneous or orthotopic tumor (stably expressing luciferase) were injected with saline, free Cy5‐siRNA, or Cy5‐siRNA/PTX(‐FA)‐LNPs (2.5 mg siRNA kg^−1^) via the tail vein. At 48 h after injection, the tumors and major organs were removed from subcutaneous tumor‐bearing mice and the livers were removed from orthotopic tumor‐bearing mice. The fluorescence or bioluminescence images were acquired using the IVIS Spectrum imaging system (PerkinElmer, MA, USA).

### Subcutaneous Xenograft Model

ICC cells (5 × 10^6^ cells per mouse) were subcutaneously injected into nude mice. The tumor size was recorded every two days, and tumor volume was calculated as follows: volume (mm^3^)  =  (*L* × *W* ^2^)/2, where *L* is the long axis and *W* is the short axis. Six weeks later, the mice were euthanized, and their tumors were isolated and weighed.

### HSC/Tumor Cell Co‐Implantation Model

ICC cells (3.5 × 10^6^ cells per mouse) and HSCs (1.5 × 10^6^ cells per mouse) were mixed and subcutaneously injected into nude mice. The tumor size was recorded every two days. The model mice were used at specific timepoints for subsequent studies.

### Lung Metastatic Model

ICC cells (5 × 10^6^ cells per mouse) were injected into the tail vein of nude mice to establish lung metastasis models. Six weeks later, tumor metastases in the lungs were visualized using the IVIS system after D‐luciferin injection. Mice were euthanized after IVIS measurement, and lungs were excised for further analysis.

### Orthotopic Tumor Model

ICC cells (5 × 10^6^ cells per mouse) were implanted into the liver of nude mice after anesthesia. Six weeks later, tumor growth was visualized using the IVIS system after D‐luciferin injection. Mice were euthanized after IVIS measurement, and livers were excised for further analysis.

### In Vivo Antitumor Effects of LNPs

To examine the effects of LNPs on tumor growth and metastasis, we established subcutaneous xenograft, lung metastasis, and orthotopic tumor models using HCCC‐9810 cells stably overexpressing cPKM. Six weeks after tumor cell transplantation, mice received different nanoformulations (2.5 mg siRNA kg^−1^) or free PTX (with an amount equal to that of PTX in siRNA/PTX‐FA‐LNPs) by injection via the tail vein. Saline was used as the control treatment. The administration schedule was twice weekly for 4 weeks.

### In Vivo Antitumor Effects of GC (Gemcitabine/Cisplatin) Combined with LNPs

To evaluate the therapeutic efficacy of GC in combination with LNPs, the HSC/tumor cell co‐implantation model was established, and the lung metastasis and orthotopic tumor models were established using HCCC‐9810 cells stably overexpressing cPKM. At the 6th week after tumor cell transplantation, different nanoformulations (2.5 mg siRNA kg^−1^) or free PTX (just for HSC/tumor cell co‐implantation model, the amount of free PTX was equal to that of PTX in siRNA/PTX‐FA‐LNPs) were applied twice a week for 4 weeks. In the 7th week, all mice except those in the control group were treated with gemcitabine (40 mg k^−1^g) and cisplatin (1 mg k^−1^g) by injection via the tail vein administered twice weekly for 3 weeks.

### Statistical Analysis

GraphPad Prism 9.0 and SPSS 23.0 were used for statistical analysis. Between‐group differences in data were assessed using Student's *t‐*test. Differences in data among three or more groups were evaluated using analysis of variance. The Mann–Whitney U test was used for comparing non‐normally distributed data. OS and RFS were assessed using the Kaplan−Meier method and compared using the log‐rank test. The χ^2^ test was used to evaluate the association between cPKM levels and clinicopathological parameters of patients. Representative data are shown as the mean ± standard deviation. A *p*‐value of <0.05 was considered to be statistically significant (*p < 0.05, **p < 0.01, ***p < 0.001), and a *p‐*value of ≥ 0.05 indicated that the differences were not significant (ns).

### Ethics Approval Statement

All clinical tissue samples were collected from the Department of Hepatobiliary Surgery, Fujian Provincial Hospital. This study was approved and authorized by the Ethics Committee of Fujian Provincial Hospital (K2021‐03‐046). All animal study procedures and experiments were approved by the Institutional Animal Care and Use Committee (IACUC) of Fujian Medical University (IACUC FJMU 2023‐Y‐0229) and were in accordance with the Guidelines for the Care and Use of Laboratory Animals.

## Conflict of Interest

The authors declare no conflict of interest.

## Author Contributions

Z.‐W.C., F.‐P.K., and C.‐K.X. contributed equally to this work. S.C., Y.‐F.T., L.H., and Z.‐W.W. were responsible for designing the research and directing the work. Z.‐W.C. and F.‐P.K. conducted the experiments and authored the paper. C.‐K.X. collected the tissues, while C.‐Y.L., G.L., and H.‐Y.L. were involved in data analysis. Y.‐D.W. and S.‐C.Z. assisted with the experiments, and J.‐F.H., C.‐F.L., and Y.H. provided technical support. All authors reviewed and approved the final manuscript.

## Supporting information

Supporting Information

## Data Availability

The data that support the findings of this study are available from the corresponding author upon reasonable request.

## References

[1] H. Sung , J. Ferlay , R. L. Siegel , M. Laversanne , I. Soerjomataram , A. Jemal , F. Bray , CA Cancer J Clin 2021, 71, 209. 33538338 10.3322/caac.21660

[2] N. Razumilava , G. J. Gores , Lancet 2014, 383, 2168. 24581682 10.1016/S0140-6736(13)61903-0 PMC4069226

[3] J. Bridgewater , P. R. Galle , S. A. Khan , J. M. Llovet , J.‐W. Park , T. Patel , T. M. Pawlik , G. J. Gores , J Hepatol 2014, 60, 1268. 24681130 10.1016/j.jhep.2014.01.021

[4] D. Moris , M. Palta , C. Kim , P. J. Allen , M. A. Morse , M. E. Lidsky , CA Cancer J Clin 2023, 73, 198. 36260350 10.3322/caac.21759

[5] I. Endo , M. Gonen , A. C. Yopp , K. M. Dalal , Q. Zhou , D. Klimstra , M. D'angelica , R. P. Dematteo , Y. Fong , L. Schwartz , N. Kemeny , E. O'Reilly , G. K. Abou‐Alfa , H. Shimada , L. H. Blumgart , W. R. Jarnagin , Ann. Surg. 2008, 248, 84. 18580211 10.1097/SLA.0b013e318176c4d3

[6] J. Valle , H. Wasan , D. H. Palmer , D. Cunningham , A. Anthoney , A. Maraveyas , S. Madhusudan , T. Iveson , S. Hughes , S. P. Pereira , M. Roughton , J. Bridgewater , N. Engl. J. Med. 2010, 362, 1273. 20375404 10.1056/NEJMoa0908721

[7] S. Rizvi , S. A. Khan , C. L. Hallemeier , R. K. Kelley , G. J. Gores , Nat. Rev. Clin. Oncol. 2018, 15, 95. 28994423 10.1038/nrclinonc.2017.157 PMC5819599

[8] R. T. Shroff , M. M. Javle , L. Xiao , A. O. Kaseb , G. R. Varadhachary , R. A. Wolff , K. P. S. Raghav , M. Iwasaki , P. Masci , R. K. Ramanathan , D. H. Ahn , T. S. Bekaii‐Saab , M. J. Borad , JAMA Oncol 2019, 5, 824. 30998813 10.1001/jamaoncol.2019.0270 PMC6567834

[9] V. Sahai , P. J. Catalano , M. M. Zalupski , S. J. Lubner , M. R. Menge , H. S. Nimeiri , H. G. Munshi , A. B. Benson , P. J. O'dwyer , JAMA Oncol 2018, 4, 1707. 30178032 10.1001/jamaoncol.2018.3277 PMC6440720

[10] A. E. Sirica , C. I. Dumur , D. J. W. Campbell , J. A. Almenara , O. O. Ogunwobi , J. L. Dewitt , Clin. Gastroenterol. Hepatol. 2009, 7, S68. 19896103 10.1016/j.cgh.2009.08.023 PMC3795391

[11] A. Moeini , D. Sia , N. Bardeesy , V. Mazzaferro , J. M. Llovet , Clin. Cancer Res. 2016, 22, 291. 26405193 10.1158/1078-0432.CCR-14-3296 PMC12224570

[12] A. E. Sirica , Nat. Rev. Gastroenterol. Hepatol. 2011, 9, 44. 22143274 10.1038/nrgastro.2011.222

[13] C. D. Fingas , S. F. Bronk , N. W. Werneburg , J. L. Mott , M. E. Guicciardi , S. C. Cazanave , J. C. Mertens , A. E. Sirica , G. J. Gores , Hepatology 2011, 54, 2076. 22038837 10.1002/hep.24588 PMC3230714

[14] N. Kang , G. J. Gores , V. H. Shah , Hepatology 2011, 54, 707. 21520207 10.1002/hep.24384 PMC3145026

[15] N. Kang , V. H. Shah , R. Urrutia , Mol. Cancer Res. 2015, 13, 604. 25548101 10.1158/1541-7786.MCR-14-0542 PMC4398610

[16] S. Memczak , M. Jens , A. Elefsinioti , F. Torti , J. Krueger , A. Rybak , L. Maier , S. D. Mackowiak , L. H. Gregersen , M. Munschauer , A. Loewer , U. Ziebold , M. Landthaler , C. Kocks , F. Le Noble , N. Rajewsky , Nature 2013, 495, 333. 23446348 10.1038/nature11928

[17] L. S. Kristensen , T. Jakobsen , H. Hager , J. Kjems , Nat. Rev. Clin. Oncol. 2022, 19, 188. 34912049 10.1038/s41571-021-00585-y

[18] J. Du , T. Lan , H. Liao , X. Feng , X. Chen , W. Liao , G. Hou , L. Xu , Q. Feng , K. Xie , M. Liao , X. Chen , J. Huang , K. Yuan , Y. Zeng , Mol Cancer 2022, 21, 18. 35039066 10.1186/s12943-021-01482-9 PMC8762882

[19] Q. Chen , H. Wang , Z. Li , F. Li , L. Liang , Y. Zou , H. Shen , J. Li , Y. Xia , Z. Cheng , T. Yang , K. Wang , F. Shen , J Hepatol 2022, 76, 135. 34509526 10.1016/j.jhep.2021.08.027

[20] A. S. Thakor , S. S. Gambhir , CA Cancer J Clin 2013, 63, 395. 24114523 10.3322/caac.21199

[21] G. Baldassarre , B. Belletti , M. S. Nicoloso , M. Schiappacassi , A. Vecchione , P. Spessotto , A. Morrione , V. Canzonieri , A. Colombatti , Cancer Cell 2005, 7, 51. 15652749 10.1016/j.ccr.2004.11.025

[22] E. Alli , J. Bash‐Babula , J. M. Yang , W. N. Hait , Cancer Res. 2002, 62, 6864. 12460900

[23] S. Singer , V. Ehemann , A. Brauckhoff , M. Keith , S. Vreden , P. Schirmacher , K. Breuhahn , Hepatology 2007, 46, 759. 17663418 10.1002/hep.21736

[24] P. Shen , T. Yang , Q. Chen , H. Yuan , P. Wu , B. Cai , L. Meng , X. Huang , J. Liu , Y. Zhang , W. Hu , Y. Miao , Z. Lu , K. Jiang , Mol Cancer 2021, 20, 51. 33750389 10.1186/s12943-021-01333-7 PMC7941935

[25] W.‐C. Liang , C.‐W. Wong , P.‐P. Liang , M. Shi , Y. Cao , S.‐T. Rao , S. K.‐W. Tsui , M. M.‐Y. Waye , Q. Zhang , W.‐M. Fu , J.‐F. Zhang , Genome Biol. 2019, 20, 84. 31027518 10.1186/s13059-019-1685-4 PMC6486691

[26] A. Datta , S. Deng , V. Gopal , K. C. Yap , C. E. Halim , M. L. Lye , M. S. Ong , T. Z. Tan , G. Sethi , S. C. Hooi , A. P. Kumar , C. T. Yap , Cancers (Basel) 2021, 13, 1882. 33919917 10.3390/cancers13081882 PMC8070945

[27] I. Mederacke , C. C. Hsu , J. S. Troeger , P. Huebener , X. Mu , D. H. Dapito , J.‐P. Pradere , R. F. Schwabe , Nat. Commun. 2013, 4, 2823. 24264436 10.1038/ncomms3823 PMC4059406

[28] S. Rizvi , G. J. Gores , Gastroenterology 2013, 145, 1215. 24140396 10.1053/j.gastro.2013.10.013 PMC3862291

[29] A. Huang , H. Zheng , Z. Wu , M. Chen , Y. Huang , Theranostics 2020, 10, 3503. 32206104 10.7150/thno.42174 PMC7069073

[30] H. Huang , H. Weng , W. Sun , X. Qin , H. Shi , H. Wu , B. S. Zhao , A. Mesquita , C. Liu , C. L. Yuan , Y.‐C. Hu , S. Hüttelmaier , J. R. Skibbe , R. Su , X. Deng , L. Dong , M. Sun , C. Li , S. Nachtergaele , Y. Wang , C. Hu , K. Ferchen , K. D. Greis , X. Jiang , M. Wei , L. Qu , J.‐L. Guan , C. He , J. Yang , J. Chen , Nat. Cell Biol. 2018, 20, 285. 29476152 10.1038/s41556-018-0045-z PMC5826585

[31] D. Hollingworth , A. M. Candel , G. Nicastro , S. R. Martin , P. Briata , R. Gherzi , A. Ramos , Nucleic Acids Res. 2012, 40, 6873. 22547390 10.1093/nar/gks368 PMC3413153

[32] N. Degrauwe , M.‐L. Suvà , M. Janiszewska , N. Riggi , I. Stamenkovic , Genes Dev. 2016, 30, 2459. 27940961 10.1101/gad.287540.116 PMC5159662

[33] R. Derynck , D. V. Goeddel , A. Ullrich , J. U. Gutterman , R. D. Williams , T. S. Bringman , W. H. Berger , Cancer Res. 1987, 47, 707. 3467839

[34] C. Liu , D. D. Billadeau , H. Abdelhakim , E. Leof , K. Kaibuchi , C. Bernabeu , G. S. Bloom , L. Yang , L. Boardman , V. H. Shah , N. Kang , J. Clin. Invest. 2013, 123, 1138. 23454766 10.1172/JCI63836 PMC3582119

[35] K. Tu , J. Li , V. K. Verma , C. Liu , D. D. Billadeau , G. Lamprecht , X. Xiang , L. Guo , R. Dhanasekaran , L. R. Roberts , V. H. Shah , N. Kang , Hepatology 2015, 61, 361. 24917558 10.1002/hep.27251 PMC4262723

[36] M. Lei , G. Zheng , Q. Ning , J. Zheng , D. Dong , Mol Cancer 2020, 19, 30. 32059672 10.1186/s12943-020-1135-7 PMC7023758

[37] J. Yu , Q.‐G. Xu , Z.‐G. Wang , Y. Yang , L. Zhang , J.‐Z. Ma , S.‐H. Sun , F. Yang , W.‐P. Zhou , J Hepatol 2018, 68, 1214. 29378234 10.1016/j.jhep.2018.01.012

[38] Z.‐Q. Hu , S.‐L. Zhou , J. Li , Z.‐J. Zhou , P.‐C. Wang , H.‐Y. Xin , L. Mao , C.‐B. Luo , S.‐Y. Yu , X.‐W. Huang , Y. Cao , J. Fan , J. Zhou , Hepatology 2020, 72, 906. 31838741 10.1002/hep.31068

[39] T. B. Hansen , T. I. Jensen , B. H. Clausen , J. B. Bramsen , B. Finsen , C. K. Damgaard , J. Kjems , Nature 2013, 495, 384. 23446346 10.1038/nature11993

[40] A. Grimson , K. K.‐H. Farh , W. K. Johnston , P. Garrett‐Engele , L. P. Lim , D. P. Bartel , Mol. Cell 2007, 27, 91. 17612493 10.1016/j.molcel.2007.06.017 PMC3800283

[41] J. Liu , J. Li , K. Wang , H. Liu , J. Sun , X. Zhao , Y. Yu , Y. Qiao , Y. Wu , X. Zhang , R. Zhang , A. Yang , Signal Transduct Target Ther 2021, 6, 42. 33526768 10.1038/s41392-020-00396-0 PMC7851151

[42] J. Trovik , E. Wik , I. M. Stefansson , J. Marcickiewicz , S. Tingulstad , A. C. Staff , T. S. Njolstad , I. Vandenput , F. Amant , L. A. Akslen , H. B. Salvesen , Clin. Cancer Res. 2011, 17, 3368. 21242118 10.1158/1078-0432.CCR-10-2412

[43] Y. Lu , P. S. Low , Adv. Drug Delivery Rev. 2002, 54, 675. 10.1016/s0169-409x(02)00042-x 12204598

[44] J. M. Campbell , T. A. Bacon , E. Wickstrom , J. Biochem. Biophys. Methods 1990, 20, 259. 2188993 10.1016/0165-022x(90)90084-p

[45] V. P. Chauhan , J. D. Martin , H. Liu , D. A. Lacorre , S. R. Jain , S. V. Kozin , T. Stylianopoulos , A. S. Mousa , X. Han , P. Adstamongkonkul , Z. Popovic , P. Huang , M. G. Bawendi , Y. Boucher , R. K. Jain , Nat. Commun. 2013, 4, 2516. 24084631 10.1038/ncomms3516 PMC3806395

[46] Z. He , X. Ruan , X. Liu , J. Zheng , Y. Liu , L. Liu , J. Ma , L. Shao , D. Wang , S. Shen , C. Yang , Y. Xue , J Exp Clin Cancer Res 2019, 38, 65. 30736838 10.1186/s13046-019-1065-7 PMC6368736

[47] L. H. Saal , P. Johansson , K. Holm , S. K. Gruvberger‐Saal , Q.‐B. She , M. Maurer , S. Koujak , A. A. Ferrando , P. Malmström , L. Memeo , J. Isola , P.‐O. Bendahl , N. Rosen , H. Hibshoosh , M. Ringnér , Å. Borg , R. Parsons , Proc Natl Acad Sci USA 2007, 104, 7564. 17452630 10.1073/pnas.0702507104 PMC1855070

[48] A. M. Karst , K. Levanon , S. Duraisamy , J. F. Liu , M. S. Hirsch , J. L. Hecht , R. Drapkin , Gynecol. Oncol. 2011, 123, 5. 21683992 10.1016/j.ygyno.2011.05.021 PMC3171572

[49] X. Liu , Y. Zhang , S. Zhou , L. Dain , L. Mei , G. Zhu , J Control Release 2022, 348, 84. 35649485 10.1016/j.jconrel.2022.05.043 PMC9644292

[50] F. Rossi , M. Beltran , M. Damizia , C. Grelloni , A. Colantoni , A. Setti , G. Di Timoteo , D. Dattilo , A. Centrón‐Broco , C. Nicoletti , M. Fanciulli , P. Lavia , I. Bozzoni , Mol. Cell 2022, 82, 75. 34942120 10.1016/j.molcel.2021.11.032 PMC8751636

[51] Z.‐W. Chen , J.‐F. Hu , Z.‐W. Wang , C.‐Y. Liao , F.‐P. Kang , C.‐F. Lin , Y. Huang , L. Huang , Y.‐F. Tian , S. Chen , J Exp Clin Cancer Res 2022, 41, 153. 35459186 10.1186/s13046-022-02343-z PMC9034615

[52] A. Du , S. Li , Y. Zhou , C. Disoma , Y. Liao , Y. Zhang , Z. Chen , Q. Yang , P. Liu , S. Liu , Z. Dong , A. Razzaq , S. Tao , X. Chen , Y. Liu , L. Xu , Q. Zhang , S. Li , J. Peng , Z. Xia , Mol Cancer 2022, 21, 109. 35524319 10.1186/s12943-022-01575-z PMC9074191

[53] H. Li , K. Peng , K. Yang , W. Ma , S. Qi , X. Yu , J. He , X. Lin , G. Yu , Theranostics 2022, 12, 6422. 36168634 10.7150/thno.77350 PMC9475446

