# Supplementary material for: A Novel Trojan Horse Nanotherapy Strategy Targeting the cPKM‐STMN1/TGFB1 Axis for Effective Treatment of Intrahepatic Cholangiocarcinoma
Source: Adv Sci (Weinh). 2023 Oct 3;10(32):2303814. doi: 10.1002/advs.202303814 (PMC10646249; doi:10.1002/advs.202303814)
Supplement: Supplementary file 1 — Supporting Information [file ADVS-10-2303814-s001.pdf]

ADVANCED SCIENCE
Open Access

Supporting Information

for *Adv. Sci.*, DOI 10.1002/advs.202303814

A Novel Trojan Horse Nanotherapy Strategy Targeting the cPKM-STMN1/TGFB1 Axis for Effective Treatment of Intrahepatic Cholangiocarcinoma

Zhi-Wen Chen, Feng-Ping Kang, Cheng-Ke Xie, Cheng-Yu Liao, Ge Li, Yong-Ding Wu, Hong-Yi Lin, Shun-Cang Zhu, Jian-Fei Hu, Cai-Feng Lin, Yi Huang, Yi-Feng Tian, Long Huang*, Zu-Wei Wang* and Shi Chen**

Supplementary Information

Supplement Figure Legends

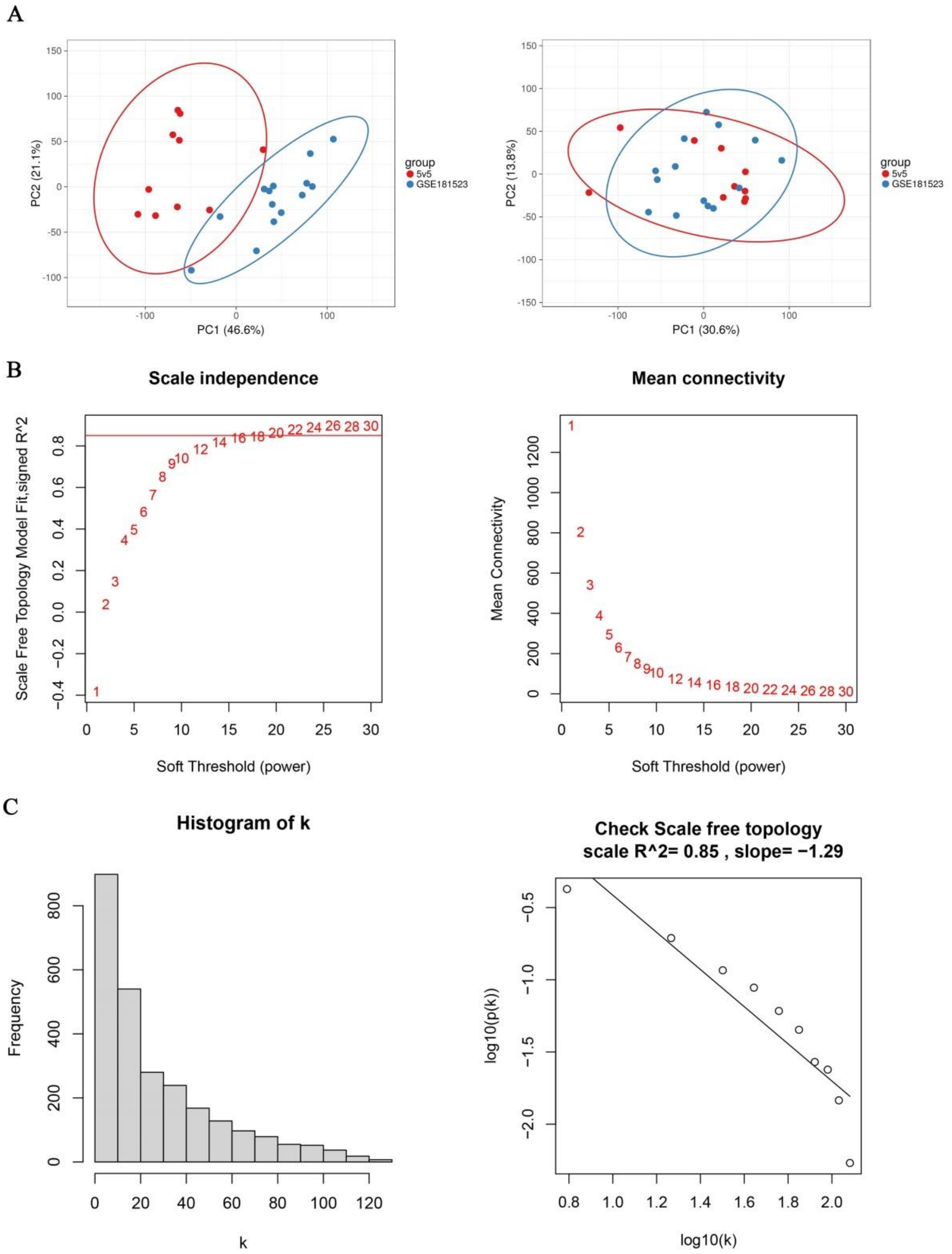

Figure S1. Batch effect correction and screening of soft threshold power in WGCNA

(A) A PCA clustering plot of two datasets before (left) and after (right) batch effect correction. **(B)** Analysis of a scale-free topology model fit parameters with different soft threshold powers (left). Analysis of mean connectivity with different soft threshold powers (right). **(C)** Histogram of connectivity distribution (left) and check of scale-free topology (right).

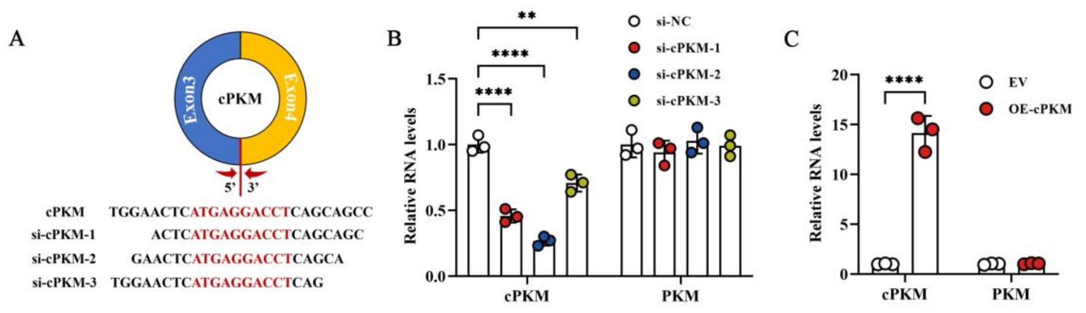

Figure S2. cPKM knockdown and overexpression validation

(A) The sequences of three siRNAs targeting the back-splicing junction of cPKM. **(B)** The levels of cPKM and PKM mRNAs were determined by qRT-PCR after the transfection of the indicated siRNAs (n = 3). **(C)** The levels of cPKM and PKM mRNAs were determined by qRT-PCR after the transfection of the indicated vectors. si-NC, negative control siRNA; si-cPKM, cPKM-targeting siRNA (n = 3). n indicates biological replicates. Data are the mean ± SD $^{**}P < 0.01$, $^{****}P < 0.0001$. Two-way ANOVA (B, C).

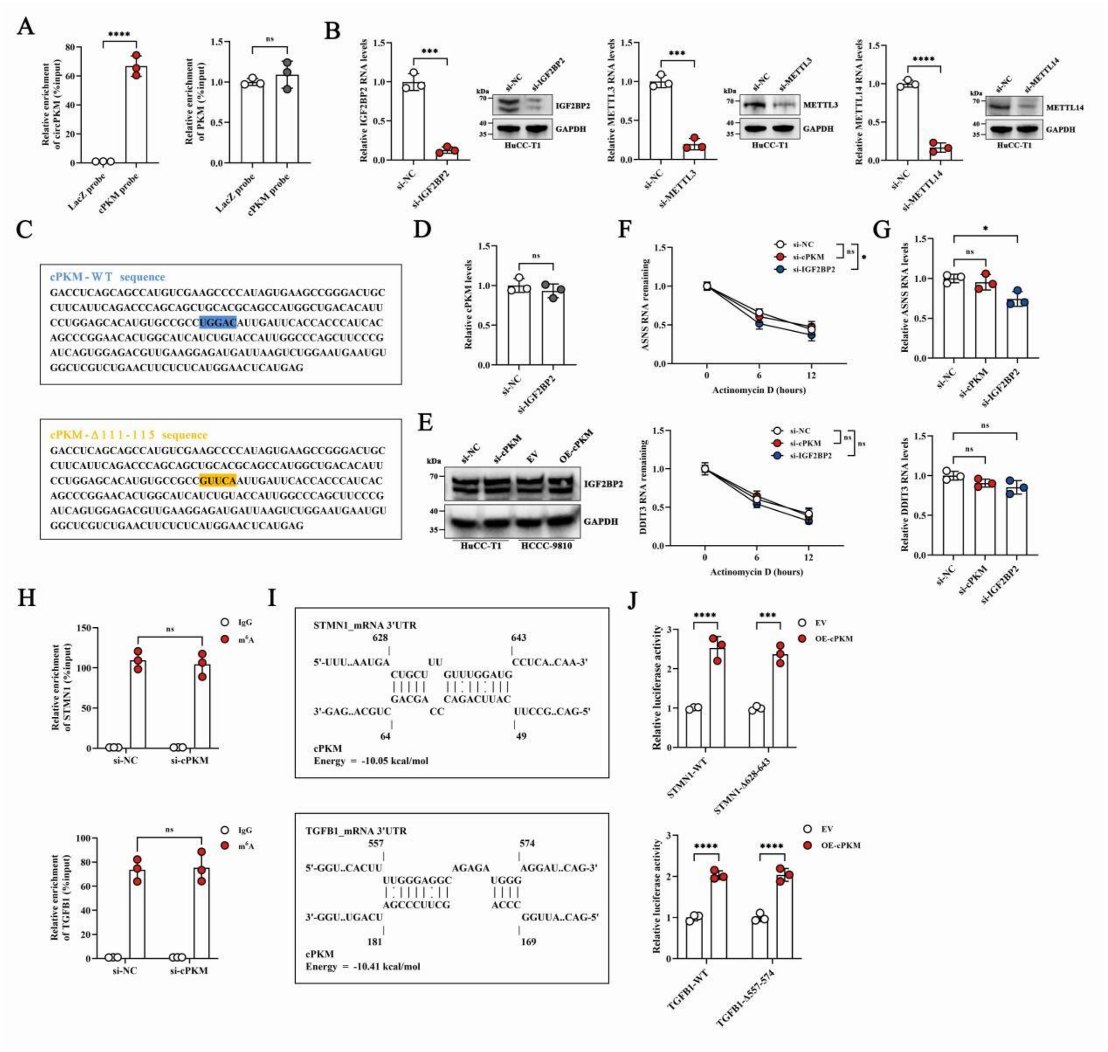

Figure S3. cPKM enrichment efficiency and knockdown efficiency of IGF2BP2, METTL3, and METTL14, and negative results associated with Figure 2

(A) cPKM and PKM levels enriched by cPKM probes in RAP assays (*n* = 3). **(B)** Validation of knockdown efficiency of IGF2BP2 (**left**), METTLE3 (**middle**), and METTLE14 (**right**) (*n* = 3). **(C)** The sequences of wild-type cPKM and mutated cPKM. **(D)** The levels of cPKM after IGF2BP2 knockdown (*n* = 3). **(E)** Protein levels of IGF2BP2 after cPKM knockdown or overexpression. **(F)** The mRNA levels of ASNS and DDIT3 were analyzed by qRT-PCR in HuCC-T1 cells with cPKM or IGF2BP2 knockdown after ActD treatment at different timepoints (*n* = 3). **(G)** mRNA levels of ASNS and DDIT3 in HuCC-T1 cells with cPKM or IGF2BP2 knockdown (*n* = 3). **(H)** The MeRIP assay to determine the level of m^6A modification in STMN1 and

TGFB1 mRNAs after cPKM knockdown (n = 3). **(I)** Potential interaction sites of cPKM with STMN1 or TGFB1 mRNA as predicted by IntaRNA. **(J)** The relative luciferase activity of the indicated luciferase reporter vectors after cPKM overexpression (n = 3). n indicates biological replicates. Data are the mean ± SD *$P < 0.05$, ***$P < 0.001$, ****$P < 0.0001$. Unpaired two-tailed Student's t-tests (A, B, D) and two-way (F, H, J) or one-way (G) ANOVA.

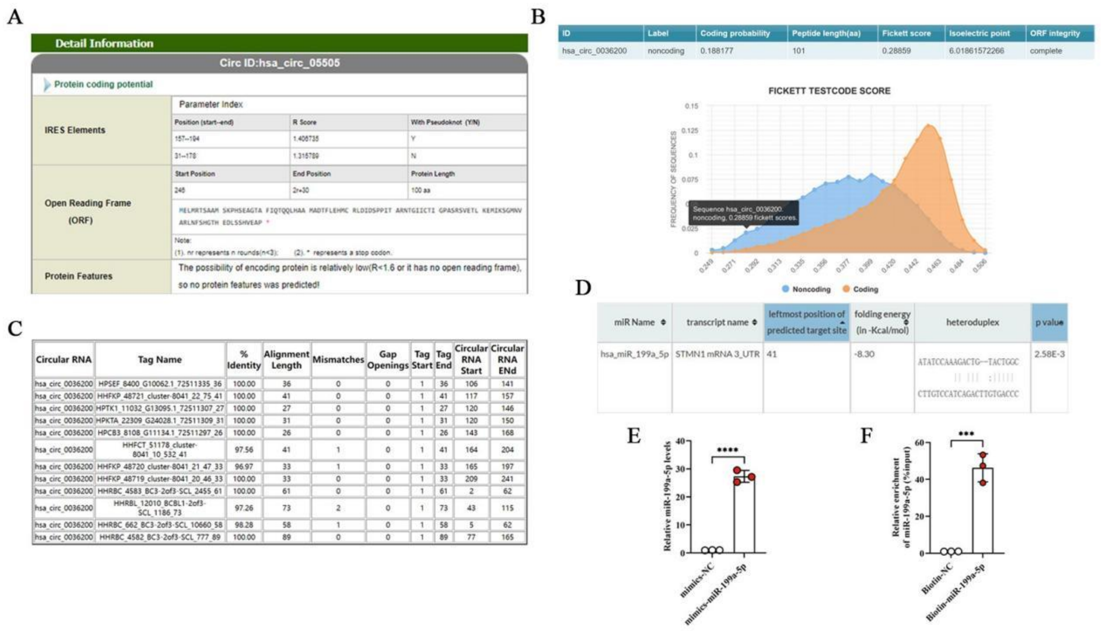

Figure S4. Bioinformatics analysis results and miR-199a-5p overexpression and enrichment efficiency

(A, B) The coding potential of cPKM was predicted on the basis of the circRNADb database **(A)** or CPC2 algorithm **(B)**. **(C)** The interaction of cPKM and AGO2 was predicted on the basis of the CircInteractome database. **(D)** The binding site of miR-199a-5p and the STMN1 mRNA 3′UTR was analyzed using the RNA22 tool. **(E)** Validation of miR-199a-5p overexpression efficiency (n = 3). **(F)** The levels of miR-199a-5p enriched by biotin-labeled miR-199a-5p mimics (n = 3). n indicates biological replicates. Data are the mean ± SD ***$P < 0.001$, ****$P < 0.0001$. Unpaired two-tailed Student's t-tests (E, F).

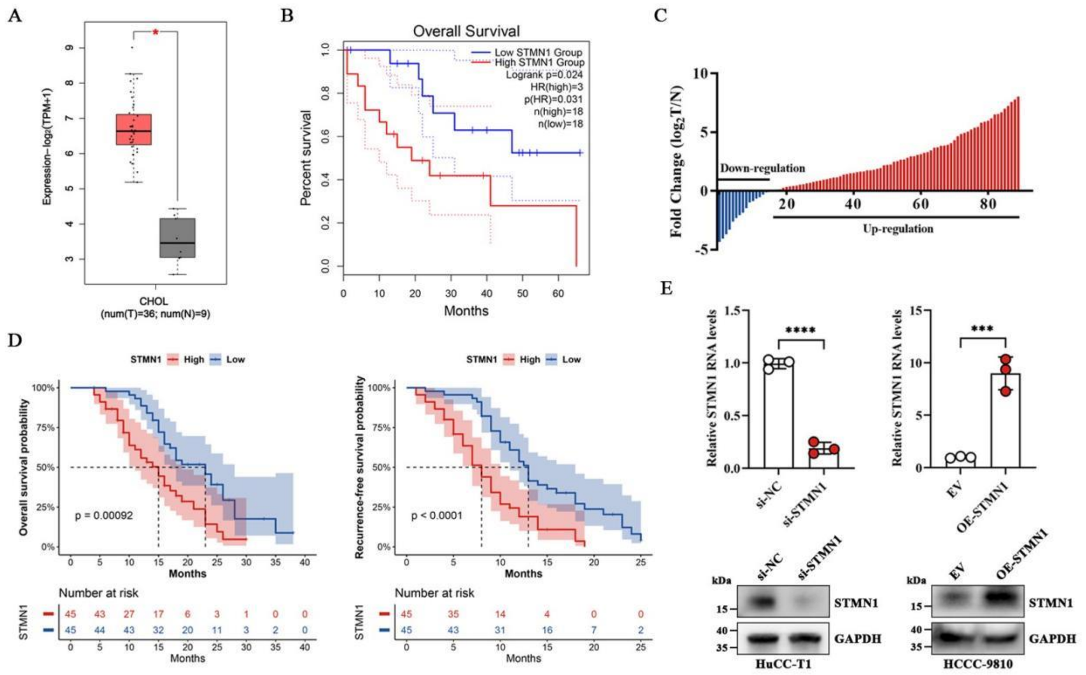

Figure S5. The expression levels of STMN1, survival curves of patients with ICC in public cholangiocarcinoma datasets, and the validation of STMN1 knockdown and overexpression efficiencies

(A) The expression levels of STMN1 between cholangiocarcinoma and normal tissues in the GEPIA2 database. **(B)** Survival curves of cholangiocarcinoma patients with low or high STMN1 expression in the GEPIA2 database. **(C)** The level of STMN1 in ICC tissues was detected using qRT-PCR. (**D**) The Kaplan–Meier survival analysis of the association of the STMN1 level with overall survival (OS) and recurrence-free survival (RFS) in 90 patients with ICC. The median STMN1 level was used as the cut-off value. **(E)** Validation of STMN1 knockdown or overexpression efficiency (n = 3). n indicates biological replicates. Data are the mean ± SD ***P < 0.001, ****P < 0.0001. Log-rank test (D) and unpaired two-tailed Student’s t-tests (E).

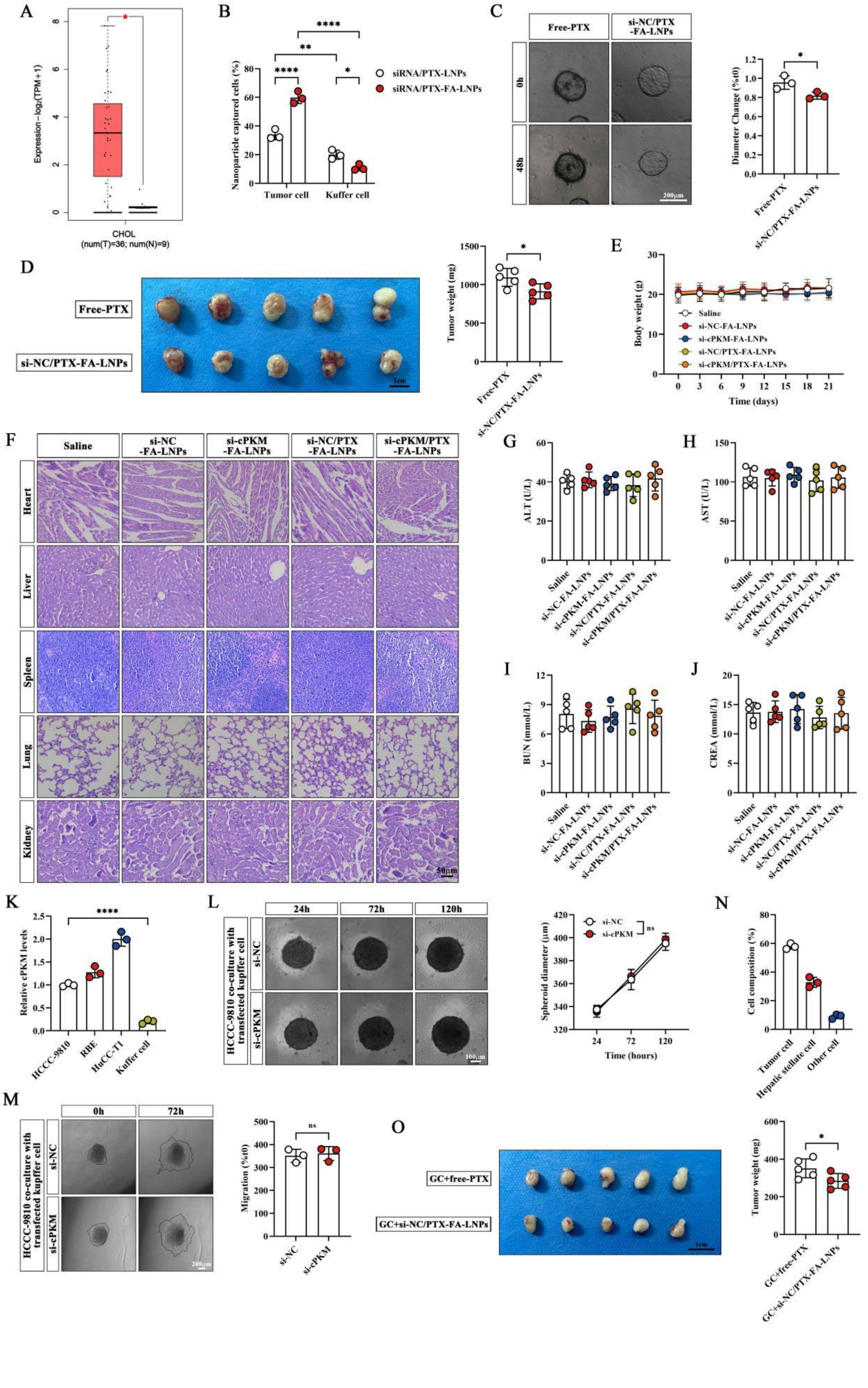

Figure S6. Additional results related to Figure 6, Figure 7, and Figure 8

(A) The difference in the expression levels of FOLR1 between cholangiocarcinoma and normal tissues in the GEPIA2 database. **(B)** Quantitative analysis of the percentage of Cy5-positive cells in Kupffer cells or tumor cells in the livers of orthotopic model mice with the indicated LNP treatment (*n* = 3). **(C)** Representative images and statistical analysis of ICC organoid growth after si-NC/PTX-FA-LNP or free PTX (with an amount equal to that of PTX in si-NC/PTX-FA-LNPs) treatment (*n* = 3). **(D)** Images and weights of subcutaneous xenografts after si-NC/PTX-FA-LNP or free PTX (with an amount equal to that of si-NC/PTX-FA-LNPs) treatment (*n* = 5). **(E)** The body weights of subcutaneous tumor-bearing mice after the indicated treatments (*n* = 5). **(F)** H&E staining of the main organs of subcutaneous tumor-bearing mice after the indicated treatments. **(G-J)** The liver function (ALT and AST levels; **G** and **H**) and kidney function (BUN and CREA levels; **I** and **J**) of subcutaneous tumor-bearing mice after the indicated treatments (*n* = 5). **(K)** The relative levels of cPKM in ICC cells and Kupffer cells were determined by qRT-PCR (*n* = 3). **(L)** Representative images of HCCC-9810 microtumor spheroids and sphere diameters after co-culture with the indicated Kupffer cells at different timepoints (*n* = 3). **(M)** Representative images of HCCC-9810 microtumor spheroid migration and the relative migration area after co-culture with the indicated Kupffer cells at different timepoints (*n* = 3). **(N)** The proportion of different cell types in the xenografts of the co-implantation model (*n* = 3). **(O)** Images and weights of subcutaneous xenografts after the indicated treatments (the amount of free PTX was equal to that of PTX in si-NC/PTX-FA-LNPs; *n* = 5). *n* indicates biological replicates (B, C, K-M) or individual mice (D, E, G-J, O). Data are the mean ± SD $^*P < 0.05$, $^{**}P < 0.01$, $^{****}P < 0.0001$. Two-way (B, E, L) or one-way (G-K) ANOVA and unpaired two-tailed Student's *t*-tests (N, M, and O).

Table S1. The hub circRNAs in the Saddlebrown module and the upregulated circRNAs

Characteristics	Number	Name
Saddlebrown_Hub	15	hsa_circ_0020353, hsa_circ_0005418, hsa_circ_0016950, hsa_circ_0023988, hsa_circ_0002545, hsa_circ_0023983, hsa_circ_0001716, hsa_circ_0078715, hsa_circ_0023984, hsa_circ_0005402, hsa_circ_0020354, hsa_circ_0000198, hsa_circ_0016979, hsa_circ_0069152, hsa_circ_0036200
DEG_Up	28	hsa_circ_0000825, hsa_circ_0091894, hsa_circ_0081069, hsa_circ_0036200, hsa_circ_0025506, hsa_circ_0091934, hsa_circ_0000745, hsa_circ_0002688, hsa_circ_0006415, hsa_circ_0020460, hsa_circ_0091419, hsa_circ_0002348, hsa_circ_0006294, hsa_circ_0004182, hsa_circ_0005507, hsa_circ_0027033, hsa_circ_0035554, hsa_circ_0025016, hsa_circ_0016956, hsa_circ_0091994, hsa_circ_0053958, hsa_circ_0005442, hsa_circ_0020397, hsa_circ_0020396, hsa_circ_0047821, hsa_circ_0003922, hsa_circ_0006635, hsa_circ_0005223

Table S2. Proteins predicted by CircInteractome, CDSC 2.0 and RBPmap databases that potentially interact with cPKM

Characteristics	Number	Name
CircInteractome	14	AGO1, AGO2, C22ORF28, DGCR8, EIF4A3, FMRP, FUS, HuR, IGF2BP1, IGF2BP2, IGF2BP3, PTB, SFRS1, ZC3H7B
CDSC 2.0	114	AGO2, PTBP1, FUS, AGO1, FBL, NOP58, NOP56, RBM10, METTL14, WTAP, UPF1, SRSF1, MOV10, AGO1-4, U2AF2, YTHDC1, RBM47, MSI2, YTHDF1, MSI1, TARDBP, TRA2A, HNRNPUL1, HNRNPA1, U2AF1, IGF2BP1, AUH, FAM120A, IGF2BP3, EIF4G2, SAFB2, GTF2F1, SND1, TROVE2, SLBP, SF3A3, TNRC6A, CSTF2T, SMNDC1, NPM1, PRPF8, DDX42, LARP7, SLTM, XRN2, CPSF6, SBDS, LSM11, YWHAG, KHDRBS1, AGO3, FXR1, TAF15, BUD13, SRSF9, DDX3X, LIN28B, GNL3, HNRNPM, DGCR8, ILF3, RBM27, FMR1, IGF2BP2, SRSF7, RBM22, FXR2, BCCIP, SRSF3, SRSF10, HNRNPC, CNBP, ACIN1, ELAVL1, AIFM1, RANGAP1, NUMA1, RNF219, VIM, ZNF184, HNRNPA2B1, LIN28, DDX54, EIF4A3, HNRNPK, ALYREF, CAPRIN1, RTCB, ZC3H7B, LIN28A, YTHDC2, YTHDF2, NUDT21, CPSF4, FIP1L1, ATXN2, CPSF7, CPSF3, EIF3G, CPSF1, SRRM4, RBM15, CSTF2, HNRNPU, METTL3, RBPMS, RBM15B, GEMIN5, PPIG, RPS11, RPS3, SERBP1, TBRG4, YBX3
RBPmap	56	A1CF, ANKHD1, BRUNOL4, BRUNOL5, CELF1, CNOT4, DAZ3, ENOX1, FMR1, FXR1, G3BP2, HNRNPA0, HNRNPA1, HNRNPF, HNRNPH1, HNRNPK, HNRNPL, HNRPLL, IGF2BP2, KHDRBS2, KHDRBS3, LIN28A, MATR3, MBNL1, NOVA1, PCBP2, PRR3, RBFOX1, RBFOX2, RBFOX3, RBM24, RBM28, RBM3, RBM41, RBM45, RBM46, RBM47, RBM5, RBM6, SAMD4A, SNRNP70, SNRPA, SRSF1, SRSF10, SRSF2, SRSF4, SRSF5, SRSF7, SRSF8, SRSF9, TARDBP, TRA2A, YBX1, YBX2, ZC3H10, ZCRB1

Table 3. Genes with significant expression changes after cPKM knockdown

Characteristics	Number	Name
Differentially expressed genes in cPKM-KD HuCC-T1 cells (|logFC| > 1.5, p-value < 0.01)	22	SERPINB10, SERPINB2, STMN1, IFI6, MMP1, EPOR, TLR2, TLR4, TGFB1, ASNS, DDIT3, EREG, AP001610.5, WTAPP1, CXCL6, MIR4665, CTD-2154I11.2, S100A9, AC078883.4, RP11-401F2.3, AC017101.10, RP11-961A15.3

Table S4. Potential target miRNAs of cPKM predicted by ENCORI, CircMine and Circ2GO databases.

Characteristics	Number	Name
ENCORI	17	hsa-miR-4262, hsa-miR-181a-5p, hsa-miR-181b-5p, hsa-miR-181c-5p, hsa-miR-181d-5p, hsa-miR-499b-5p, hsa-miR-6509-3p, hsa-miR-199a-5p, hsa-miR-199b-5p, hsa-miR-613, hsa-miR-1-3p, hsa-miR-206, hsa-miR-5047, hsa-miR-1301-3p, hsa-miR-605-3p, hsa-miR-2682-5p, hsa-miR-34b-5p
CircMine	26	hsa-miR-199a-5p, hsa-miR-181a-2-3p, hsa-miR-1301-3p, hsa-miR-449c-5p, hsa-miR-889-5p, hsa-miR-1322, hsa-miR-2682-5p, hsa-miR-3157-5p, hsa-miR-320e, hsa-miR-4255, hsa-miR-3675-5p, hsa-miR-3692-5p, hsa-miR-3714, hsa-miR-3937, hsa-miR-1587, hsa-miR-4640-5p, hsa-miR-4675, hsa-miR-4741, hsa-miR-4790-3p, hsa-miR-5047, hsa-miR-6771-5p, hsa-miR-6777-5p, hsa-miR-6837-3p, hsa-miR-7160-5p, hsa-miR-7843-5p, hsa-miR-7974
Circ2GO	27	hsa-miR-3944-5p, hsa-miR-425-3p, hsa-miR-1-3p, hsa-miR-199b-5p, hsa-miR-449c-5p, hsa-miR-181d-5p, hsa-miR-6837-3p, hsa-miR-150-3p, hsa-miR-449b-3p, hsa-miR-181c-5p, hsa-miR-181a-2-3p, hsa-miR-93-3p, hsa-miR-676-5p, hsa-miR-34b-5p, hsa-miR-143-5p, hsa-miR-181a-5p, hsa-miR-6509-3p, hsa-miR-1301-3p, hsa-miR-664a-3p, hsa-miR-3157-5p, hsa-miR-5000-5p, hsa-miR-181b-5p, hsa-miR-199a-5p, hsa-miR-1180-3p, hsa-miR-605-3p, hsa-miR-3180-5p, hsa-miR-889-5p

Table S5. The sequences of primers and oligonucleotides used in this study

Name	Sequence (5'- 3')
**Primers for qRT-PCR**	
cPKM forward	CTCATGGAACTCATGAGGAC
cPKM reverse	GTGCTCCAGGAATGTGTCAG
PKM forward	TGACGGAGGTGGAAAATGGTG
PKM reverse	TGGATGTCCTTCTCCGACAC
STMN1 forward	TGATTCTCAGCCCTCGGTCA
STMN1 reverse	GCTTCATGGGACTTGCGTCT
TGFB1 forward	GAGCCCTGGACACCAACTAT
TGFB1 reverse	AAGTTGGCATGGTAGCCCTT
ASAN forward	CTCCGCGCAGATCGAACTAC
ASAN reverse	TCTTTTGGTCGCCAGAGAATC
DDIT3 forward	CTGGAAAGCAGCGCATGAAG
DDIT3 reverse	GGTGCAGATTCACCATTCGG
METTL3 forward	CTGCAACGCATCATTCGGAC
METTL3 reverse	AGACCCTGGTTGAAGCCTTG
METTL14 forward	TGGACCTTGGAAGAGTGTGTT
METTL14 reverse	GTGCTACGCTTCACAGTTCC
miR-199a-5p forward	ACACTCCAGCTGGGCCCAGTGTTCAGACTAC
miR-199a-5p reverse	TGGTGTCGTGGAGTCG
miR-199a-5p stem-loop	CTCAACTGGTGTCGTGGAGTCGGCAATTCAGTTGAGGA ACAGGT
miR-1301-3p forward	ATCTTGCAGCTGCCTGGGAGT
miR-1301-3p reverse	AGTGCAGGGTCCGAGGTATT
miR-1301-3p stem-loop	GTCGTATCCAGTGCAGGGTCCGAGGTATTCGCACTGGA TACGACGAAGTC
U6 forward	CTCGCTTCGGCAGCACA
U6 reverse	AACGCTTCACGAATTTGCGT
GAPDH forward	ACATCATCCCTGCCTCTACTG
GAPDH reverse	CCTGCTTCACCACCTTCTTG
**siRNAs**	
si-cPKM	GAACTCATGAGGACCTCAGCA
si-IGF2BP2	AGCGCAAGATCAGGGAAATTG

si-METTL3	GCAGTTCCTGAATTAGCTACA
si-METTL14	GGATGAGTTAATAGCTAAATC
si-STMN1	AACTGGAACGTTTGCGAGAGA
**FISH probe**	
cPKM	CTGCTGAGGTCCTCATGAGTTCCAT
miR-199a-5p	GAACAGGTAGTCTGAACACTGGG
**RAP probe**	
cPKM	CTATGGGGCTTCGACATGGCTGCTGAGGTCCTCATGAG TTCCATGAGAGAAGTTCAGACG

Table S6. Primary antibodies used in this study.

Name	Source	Identifier
IGF2BP2	Proteintech	11601-1-AP
STMN1	Abcam	ab52630
TGFB1	Huabio	HA721143
E-cadherin	Proteintech	20874-1-AP
N-cadherin	Proteintech	22018-1-AP
Vimentin	Proteintech	10366-1-AP
a-SMA	Huabio	ET1607-53
Collagen I	Proteintech	67288-1-Ig
Fibronectin	Huabio	ET1702-25
AKT	Huabio	ET1609-51
p-AKT(Ser437)	Huabio	ET1607-73
p-AKT(Thr308)	Abclonal	AP1259
METTL3	Proteintech	15073-1-AP
METTL14	Proteintech	26158-1-AP
Cytokeratin 19	Abcam	ab52625
CD34	Abcam	ab81289
HIF1a	Abcam	ab51608
Ki-67	Abcam	ab16667
GAPDH	Abcam	ab8245
